# Supplementary material for: Simplifying the screening of gestational diabetes by maternal age plus fasting plasma glucose at first prenatal visit: A prospective cohort study
Source: PLoS One. 2020 Aug 20;15(8):e0237224. doi: 10.1371/journal.pone.0237224 (PMC7444589; doi:10.1371/journal.pone.0237224)
Supplement: S1 Table — (DOCX) [file pone.0237224.s001.docx]

S1 Table. Clinical characteristics and laboratory test results at the first prenatal visit and OGTT result at 24-28 gestational weeks in pregnancy women with and without FPG and HbA1c at the first prenatal visit.

| Baseline characteristics | **With** FPG and HbA1c at the FPV | **Without** FPG and HbA1c at the FPV | P value |
| --- | --- | --- | --- |
| N | 512 | 407 |  |
| Age (years) | 33.8 (4.2) | 34 (4) | 0.36 |
| Age≥35 (N, %) | 232 (45.3 %) | 180 (44.2 %) | 0.63 |
| Nulliparous (N, %) | 168 (32.8 %) | 163 (40 %) | 0.68 |
| Gestational age at the FPV (weeks) | 10.1 (2) | 10.9 (2.6) | 0.81 |
| Family history of DM (N, %) | 122 (23.8 %) | 79 (19.4 %) | 0.11 |
| History of PCOS | 19 (3.7 %) | 14 (3.4 %) | 0.83 |
| History of macrosomia | 4 (0.8 %) | 2 (0.5 %) | 0.59 |
| Pre-pregnancy BW (kg) | 55.6 (9.5) | 56.2 (9.2) | 0.34 |
| Pre-pregnancy BMI (kg/m2) | 22 (3.7) | 22 (3.3) | 0.89 |
| GWG at 24-28 gestational weeks (kg) | 6.3 (3.5) | 6.7 (3.3) | 0.07 |
| Glucose level during OGTT at 24-28 gestational weeks | | | |
| FPG during OGTT (mg/dL) | 79.2 (7) | 78.6 (5.5) | 0.13 |
| 1hPG (mg/dL) | 133.9 (29) | 133.6 (28.2) | 0.86 |
| 2hPG (mg/dL) | 115.5 (24.9) | 115.8 (25.8) | 0.86 |

Mean (standard deviations) or N (%) were shown.

BMI, body mass index; BW, body weight; DM, diabetes mellitus; FPG, fasting plasma glucose; FPV, first prenatal visit; GDM, gestational diabetes mellitus; GWG, gestational weight gain; HbA1c, hemoglobin A1c; OGTT, oral glucose tolerance tests; FPG during OGTT, fasting plasma glucose during oral glucose tolerance tests; PCOS, Polycystic ovary syndrome; 1hPG, 1-hour plasma glucose during oral glucose tolerance tests; 2hPG, 2-hour plasma glucose during oral glucose tolerance tests
