# Supplementary material for: Simplifying the screening of gestational diabetes by maternal age plus fasting plasma glucose at first prenatal visit: A prospective cohort study
Source: PLoS One. 2020 Aug 20;15(8):e0237224. doi: 10.1371/journal.pone.0237224 (PMC7444589; doi:10.1371/journal.pone.0237224)
Supplement: S2 Table — (DOCX) [file pone.0237224.s002.docx]

S2 Table. The relationship between clinical characteristics at the first prenatal visit and of gestational diabetes mellitus (GDM) at early pregnancy. Adjusted odds ratios (OR), 95% confidence intervals (95% CI) and p values in multivariate logistic regression models are shown. GDM was used as the dependent variable, and age, fasting plasma glucose, HbA1c, family history of DM, and pre-pregnancy BMI were used as the independent variables.

|  | OR (95% CI) | p |
| --- | --- | --- |
| Age (years) | 1.1 (1.01-1.2) | **0.03** |
| FPG at FPV (mg/dL) | 1.2 (1.2-1.3) | **<0.001** |
| HbA1c (%) | 2.3 (0.7-6.8) | 0.16 |
| Family history of DM | 1.2 (0.6-2.3) | 0.49 |
| Pre-pregnancy BMI (kg/m2) | 1.0 (0.9-1.1) | 0.81 |

BMI, body mass index; DM diabetes mellitus; FPG, fasting plasma glucose; FPV, first prenatal visit; HbA1c, hemoglobin A1c.
