# Supplementary material for: Simplifying the screening of gestational diabetes by maternal age plus fasting plasma glucose at first prenatal visit: A prospective cohort study
Source: PLoS One. 2020 Aug 20;15(8):e0237224. doi: 10.1371/journal.pone.0237224 (PMC7444589; doi:10.1371/journal.pone.0237224)
Supplement: S3 Table — (DOCX) [file pone.0237224.s003.docx]

S3 Table. The 2x2 table illustrating the calculation of the sensitivity and the specificity for "the FPG at the FPV algorithm" with the cutoff at 79 mg/dl.

|  | **GDM +**  by the IADPSG criteria | **GDM-**  by the IADPSG criteria | Total |
| --- | --- | --- | --- |
| GDM diagnosed by the algorithm | 69 | 0 | 69 |
| GDM excluded by the algorithm | 6 | 437 | 443 |
| Total | 75 | 437 | 512 |
| Sensitivity | 69/75 = 0.92 |  |  |
| Specificity | 437/437 = 1.00 |  |  |
