# Supplementary material for: Simplifying the screening of gestational diabetes by maternal age plus fasting plasma glucose at first prenatal visit: A prospective cohort study
Source: PLoS One. 2020 Aug 20;15(8):e0237224. doi: 10.1371/journal.pone.0237224 (PMC7444589; doi:10.1371/journal.pone.0237224)
Supplement: S1 Fig — (DOCX) [file pone.0237224.s004.docx]

S1 Fig. Fasting plasma glucose (FPG) at the first prenatal visit by age group. * p for trend across all age groups <0.05.


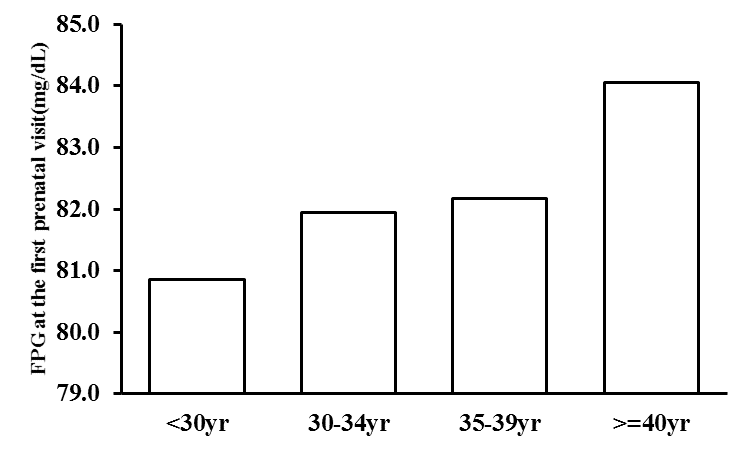


*
