# Supplementary material for: Simplifying the screening of gestational diabetes by maternal age plus fasting plasma glucose at first prenatal visit: A prospective cohort study
Source: PLoS One. 2020 Aug 20;15(8):e0237224. doi: 10.1371/journal.pone.0237224 (PMC7444589; doi:10.1371/journal.pone.0237224)

S2 Fig. Algorithms to screen gestational diabetes mellitus (GDM) by (A) fasting plasma glucose (FPG) at the first prenatal visit (FPV) and (B) age plus FPG at the FPV. The number of pregnant women in different paths are shown. The unit for age is years and the unit for FPG is mg/dL. OGTT, oral glucose tolerance tests; GDM excluded, pregnant women who were diagnosed as not having GDM; GDM diagnosed, pregnant women who were diagnosed as GDM.


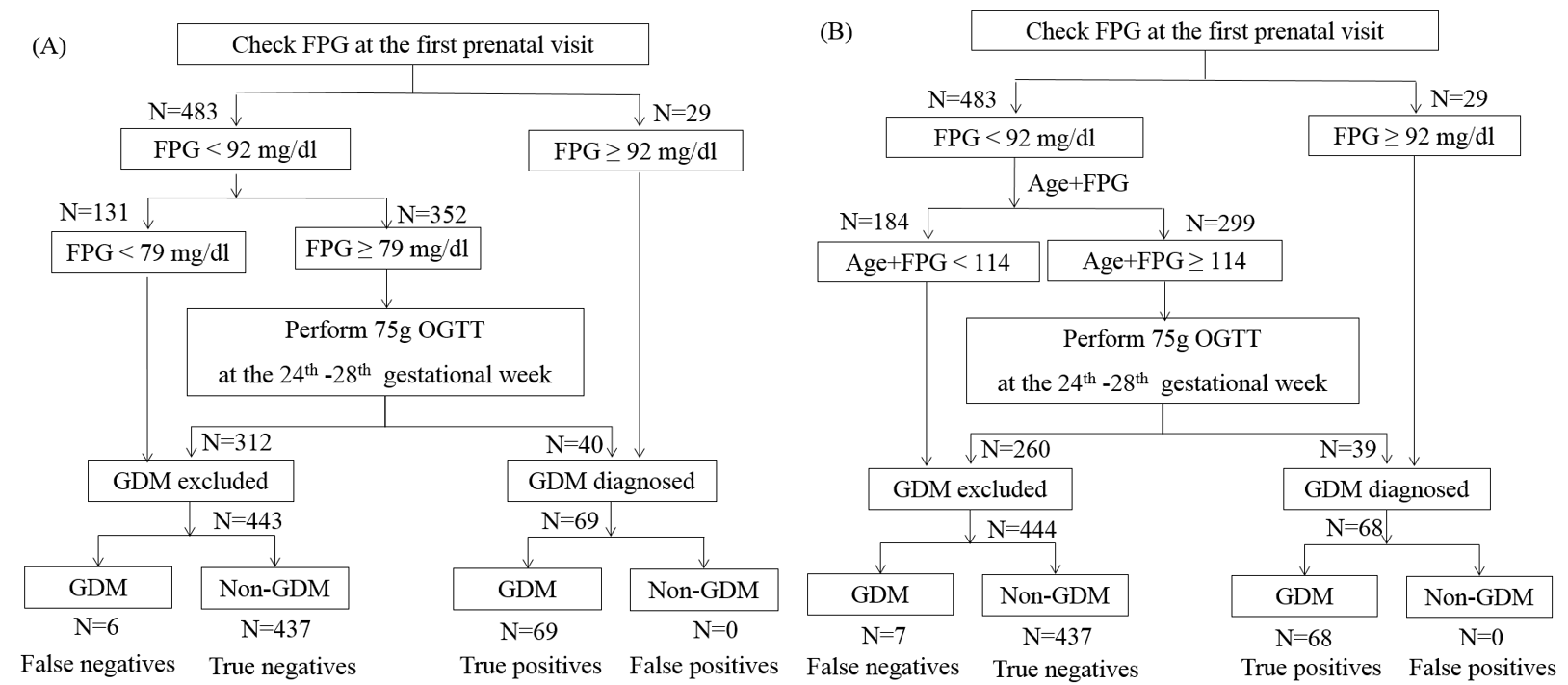

Supplement: S2 Fig — Algorithms to screen gestational diabetes mellitus (GDM) by (A) fasting plasma glucose (FPG) at the first prenatal visit (FPV) and (B) age plus FPG at the FPV. (DOCX) [file pone.0237224.s005.docx]
