# Supplementary material for: Simplifying the screening of gestational diabetes by maternal age plus fasting plasma glucose at first prenatal visit: A prospective cohort study
Source: PLoS One. 2020 Aug 20;15(8):e0237224. doi: 10.1371/journal.pone.0237224 (PMC7444589; doi:10.1371/journal.pone.0237224)
Supplement: Attachment — Submitted filename: Response to Reviewers.docx [file pone.0237224.s006.docx]

Manuscript No.: PONE-D-20-00081

Title: Simplifying the gestational diabetes screen by maternal age plus fasting plasma glucose at first prenatal visit: a prospective cohort study

PLOS ONE

Responses to Reviewer #1:

Thank you very much for your constructive suggestions for this manuscript. We have made the revisions according to the comments, point by point, as follows. The page and line are according to revised manuscript with track changes.

1. “The international diabetes study groups, including the International Association of Diabetes and Pregnancy Study groups (IADPSG) and the American Diabetes Association(ADA) have adopted the 75-g oral glucose tolerance test (OGTT) at 24–28 weeks of gestation as a screening test for gestational diabetes mellitus (GDM).” If you mention ADA and IADPSG, the correct reference must be reported both in the text and in the bibliography. In the references IADPSG has been reported with number 12.

*Ans*: Thanks for your question. We have revised the manuscript according to your suggestion.

In Introduction (page 5, line 59-64): For the diagnosis of gestational diabetes mellitus (GDM), the International Association of Diabetes and Pregnancy Study groups (IADPSG) and the World Health Organization (WHO) recommends the use of 75-g oral glucose tolerance test (OGTT) for all pregnant women at 24–28 weeks of gestation, which is also a diagnostic option suggested by the American Diabetes Association (ADA) in addition to the two-step method **(1-3)**.

In Reference:

1.	International Association of D, Pregnancy Study Groups Consensus P, Metzger BE, Gabbe SG, Persson B, Buchanan TA, et al. International association of diabetes and pregnancy study groups recommendations on the diagnosis and classification of hyperglycemia in pregnancy. Diabetes Care. 2010;33(3):676-82.

2.	Diagnostic Criteria and Classification of Hyperglycaemia First Detected in Pregnancy. WHO Guidelines Approved by the Guidelines Review Committee. Geneva2013.

3.	American Diabetes A. 2. Classification and Diagnosis of Diabetes: Standards of Medical Care in Diabetes-2020. Diabetes Care. 2020;43(Suppl 1):S14-S31.

2. “However, these FPG-based screening methods have generally been targeted at 24–28 weeks of gestation instead of early pregnancy, and the use of an FPG-based method at 24-28 weeks essentially requires two steps to diagnose or exclude DM.” Please report the references for this sentence.

*Ans*: We have revised the manuscript according to your suggestion.

In Introduction (page 5, line 68-71): However, these FPG-based screening methods have generally been targeted at 24–28 weeks of gestation instead of early pregnancy, and the use of an FPG-based method at 24-28 weeks essentially requires two steps to diagnose or exclude DM **(5-8).”**

3. “On the other hand, FPG is nowadays routinely measured during early pregnancy to detect pre-existing diabetes according to the IADPSG and ADA (4) diagnostic protocols.” Please check the references and position them at the end of the sentence.

*Ans*: We have revised the manuscript according to your suggestion.

In Introduction (page 5-6, line 72-76): On the other hand, the IADPSG recommends the measurement of FPG during early pregnancy to detect pre-existing diabetes and GDM **(1, 9)**; whereas the ADA suggests the screening of undiagnosed type 2 diabetes in the first prenatal visit (FPV) in women with risk factors by the standard criteria, such as FPG, hemoglobin A1c or even OGTT **(3)**.

4. “As such, the FPG level at the FPV, which is easy to obtain, may be a good predictor to be used in screening GDM.” Please report the references for this sentence.

*Ans*: We have revised the manuscript according to your suggestion.

In Introduction (page 6, line 75-78): Since higher first-trimester FPG levels are associated with an increased risk of GDM diagnosed at 24-28 weeks **(10)**, the FPG at the FPV may be a good predictor to be used in the screening of GDM.

5. “Aging is thought to be one of the most important factors affecting the pathogenic mechanisms associated with diabetes (6). However, despite the widely reported fact that increasing age increases the prevalence of diabetes, information regarding the relationship between age and GDM is reported relatively rarely. Furthermore, advanced maternal age is also a growing trend. In most developed countries, women get pregnant at older ages (7-10). Therefore, maternal age may be another important factor to be considered in GDM screening, especially when women become pregnant at an older age.” This paragraph does not seem to be written in correct English, please review the English grammar and the connection between the various sentences.

*Ans*: Thanks for your suggestion. We have revised the manuscript according to your suggestions as below.

In Introduction (page 6, line 80-84): Although increasing maternal age is known to be a significant risk factor of diabetes mellitus (DM) (11), information regarding the relationship between age and GDM is reported relatively rarely. In most developed countries, women get pregnant at older ages (12-15). Therefore, maternal age may be another important factor to be considered in the screening of GDM, especially in countries where women become pregnant at an older age.

6. It is unclear how women with a blood glucose value ≥ 92 mg/dl are managed in the first trimester. OGTT has also been performed in these women? According to the IADPSG guidelines, if the value is ≥ 92 and <126 mg/dl a diagnosis of gestational diabetes must be made and treated for this complication. What treatment were they subjected to? Did they perform OGTT even if in therapy? If OGTT has been performed on women already on therapy this could be an important bias in your study.

*Ans*: Thanks for your suggestion. In this study, pregnant women in the first trimester with fasting blood glucose value ≥ 92 mg/dl and <126 mg/dl were diagnosed as gestational diabetes according to the IADPSG guidelines. They were treated and did not receive OGTT in the second trimester. Only the women with the 1^st^ trimester fasting plasma glucose (FPG) < 92 mg/dl received OGTT. We have revised our manuscript to make it clearer.

In Research design and methods (page 7-8, line 107-112): Eligible participants received blood tests for FPG and haemoglobin A1c (HbA1c) at the FPV after fasting for 8-10 hours. Women with FPG at the FPV less than 92 mg/dL received 75-g OGTT at 24-28 gestational weeks. The diagnosis of GDM was based on the recommendations of the IADPSG if FPG at the FPV ≥92 mg/dL, or if one of the results from the OGTT exceeded the cutoffs, including FPG ≥92 mg/dL, plasma glucose at 1h ≥180 mg/dL, or plasma glucose at 2h ≥153 mg/dL (1).

7. “Maternal blood samples were collected after 8–10 hours of overnight fasting
during and were used to determine HbA1c and FPG.” In which trimester was this maternal blood samples performed? This sentence is unclear and seems to be redundant. Please clarify.

*Ans:* We agree with you and have deleted this redundant sentence.

8. Personally, I find impossible to interpret the results as interesting as they seem. Text, tables and captions have been put together. Please rephrase the results completely and put them in an understandable way and following the submission guidelines.

*Ans:* We have rechecked the format requirement of the journal and confirmed that the journal requested that tables and figure legends in the manuscript must be placed directly after the paragraph in which it is first cited.

9. “A total of 547 patients were enrolled in the present study. A flowchart of the study 120 population is shown in S1 Figure.” It is not clear from the description how the cohort was selected from the general population that after to your Institute. I assume that more than 1065 women have been visited between January 2013 and June 2018. Was this a convenience sample? Were women recruited on certain days? This needs to be clarified as this a source of unrecognized bias that would need to be highlighted in the limitations.

*Ans*: In this study, study subjects were enrolled consecutively by two doctors (Shin-Yu Lin and Chien-Nan Lee). Some women who fulfilled the inclusion and exclusion criteria refused to join the study. There is no selection of study subjects. We have revised the manuscript to make it clearer. The S1 figure was re-numbered to Fig 1 in the revised version.

In Result (page 10-11, line 155-169): A total of 1065 women who had their FPV between January 2013 and June 2018 were enrolled consecutively in the training cohort and another 182 women were included in the validation cohort from January 2019 to December 2019. The standard procedures in these cohorts were as follows. 1. FPG and HbA1c were measured at FPV to diagnose pre-existing diabetes (defined as FPG ≥126 mg/dL or HbA1c ≥ 6.5% at FPV) and GDM (FPG at FPV≥92 mg/dl). 2. In women without pre-existing diabetes and GDM, OGTT was performed at 24-28 gestational weeks. Of the 1065 women who agreed to join our research, 55 women were excluded because of twin pregnancies, history of GDM, or pre-existing diabetes. We also excluded 419 women who were recruited at the second trimester and their FPG at the FPV could not be obtained. Another 79 women were excluded because they received OGTT in other clinic or hospital and the data were not available. As a result, 512 women were included in our study. A flowchart of the study population is shown in Figure 1. Besides, another 182 women were included from January 2019 to December 2019 as the validation cohort. All the study subjects in the training and validation cohorts were Chinese Han.

Fig 1. The flow chart for the inclusion and exclusion of the study population.

10. Usually the tables must be presented in an attachment to the paper and not within the text. Please check the submission methods for this journal. Also, personally I find the "All" column useless

*Ans:* As described in question #8, the journal requested that each table should be placed directly after the paragraph in which it is first cited. Besides, we have deleted the column of “All” according to your suggestions. The revised Table 1 was shown below.

In Result (page 12-13, line 184-191):

Table 1. Clinical characteristics and laboratory test results at the first prenatal visit and OGTT result at 24-28 gestational weeks in pregnancy women with and without gestational diabetes mellitus (GDM) in the training cohort and validation cohort.

|  | The training cohort | | | The validation cohort | | |
| --- | --- | --- | --- | --- | --- | --- |
| Baseline characteristics | Non-GDM | GDM | P value | Non-GDM | GDM | P value |
| N | 437 (85.4 %) | 75 (14.6 %) | NA | 125 (80.2 %) | 26 (17.2 %) | NA |
| Age (years) | 33.5 (4.1) | 35.1 (4.1) | **0.002** | 33.3 (3.6) | 33.8 (2.9) | 0.55 |
| Age≥35 (N, %) | 190 (43.5 %) | 42 (56 %) | **0.04** | 46 (36.8 %) | 11 (42.3 %) | 0.32 |
| Nulliparous (N, %) | 142 (32.5 %) | 26 (34.7 %) | 0.68 | 46 (36.8 %) | 13 (50 %) | 0.48 |
| Gestational age at the FPV (weeks) | 10.1 (1.9) | 10.3 (2.2) | 0.81 | 10 (1.5) | 9.9 (1.5) | 0.58 |
| Family history of DM (N, %) | 96 (22 %) | 26 (34.7 %) | **0.02** | 21 (16.8 %) | 5 (19.2 %) | 0.81 |
| History of PCOS | 16 (3.7 %) | 3 (4 %) | 0.89 | 7 (5.6 %) | 3 (11.5 %) | 0.27 |
| History of macrosomia | 2 (0.5 %) | 2 (2.7 %) | 0.05 | 0 | 0 | - |
| Pre-pregnancy BW (kg) | 55.3 (8.4) | 57.8 (14) | **0.03** | 55.2 (8.1) | 60.2 (8.5) | **0.005** |
| Pre-pregnancy BMI (kg/m2) | 21.9 (3.3) | 22.9 (5.3) | **0.02** | 21.9 (3.2) | 23.7 (3.5) | **0.02** |
| GWG at 24-28 gestational weeks (kg) | 6.3 (3.7) | 6.3 (2.7) | 0.4 | 6.1 (3.2) | 6.2 (2.3) | 0.92 |
| Laboratory test results at the first prenatal visit | | | |  |  |  |
| FPG (mg/dL) | 81.1 (5) | 88 (7.4) | **<0.001** | 82.8 (4.7) | 90.9 (7.2) | **<0.001** |
| HbA1c (%) | 5.2 (0.2) | 5.4 (0.3) | **<0.001** | 5.1 (0.3) | 5.3 (0.3) | **<0.001** |
| Glucose level during OGTT at 24-28 gestational weeks | | | |  |  |  |
| FPG during OGTT (mg/dL) | 77.9 (5) | 82.3 (6.9) | **<0.001** | 78.8 (5) | 85.2 (5.4) | **<0.001** |
| 1hPG (mg/dL) | 128.2 (24.3) | 164.7 (29.5) | **<0.001** | 127.6 (27.3) | 149 (34.3) | **<0.001** |
| 2hPG (mg/dL) | 109.9 (19.9) | 150.2 (29.4) | **<0.001** | 108.6 (21.3) | 135.6 (39.7) | **<0.001** |

Mean (standard deviations) or N (%) were shown. BMI, body mass index; BW, body weight; DM, diabetes mellitus; FPG, fasting plasma glucose; FPV, first prenatal visit; GDM, gestational diabetes mellitus; GWG, gestational weight gain; HbA1c, hemoglobin A1c; OGTT, oral glucose tolerance tests; FPG during OGTT, fasting plasma glucose during oral glucose tolerance tests; PCOS, Polycystic ovary syndrome; 1hPG, 1-hour plasma glucose during oral glucose tolerance tests; 2hPG, 2-hour plasma glucose during oral glucose tolerance tests

11. “As shown in Figure 1, the prevalence of GDM increased with age. Fig 1. Prevalence of gestational diabetes mellitus (GDM) by age group. * p for trend<0.001.” This sentence at this point in the text has no meaning.

*Ans*: The journal requested that figure legends should be placed directly after the paragraph in which it is first cited. To make it clearer, we have revised the paragraph. (Figure 1 in the old version is labeled as Figure 2 in the revised version.)

In Result (page 14, line 192-199): As shown in Figure 2, the incidence of GDM increased with age. The incidence of GDM was 8.9% for the age group below 30 years, 15.4% for the age group 30-34 years, 20.2% for the age group 35-39 years, and 34.8% for the age group above 40 years. In addition, the level of FPG at the FPV also increased slightly with age (S1 Figure). The FPG at FPV was also significantly higher in women with old age.

Fig 2. Incidence of gestational diabetes mellitus (GDM) by age group. * p for trend across all ages <0.001.

12. In my opinion, some important factors are missing in interpreting the results: ethnicity, weight gain on the first visit and weight gain at the time of the OGTT.
If the weight gained during pregnancy is not available, please specify it.
Is it not possible that the women with increased fasting plasma glucose in 1st trimester had a superior weight gain compared to the other groups? Also, adding GWG to the multivariate model has clinical plausibility. Also specify whether the women were of different or equal ethnic groups. This factor influenced the possibility of developing gestational diabetes.

*Ans*: All the study subjects were Han Chinese. For gestational weight gain (GWG), GWG at the time of the OGTT was available but GWG at the first visit was not available in the study. As shown in the following Table A, GWG at the time of the OGTT was not a significant predictor of GDM in this study. Since Table S2 only included variables available in the first prenatal visit, we did not include GWG at the time of the OGTT in the model. We have revised the manuscript to provide the information.

Table A. The relationship between clinical characteristics at the first prenatal visit and of gestational diabetes mellitus (GDM) at early pregnancy. Adjusted odds ratios (OR), 95% confidence intervals (95% CI) and p values in multivariate logistic regression models are shown. GDM was used as the dependent variable, and age, fasting plasma glucose, HbA1c, Family history of DM, Pre-pregnancy BMI, and GWG at the time of the OGTT were used as the independent variables.

|  | OR (95% CI) | p |
| --- | --- | --- |
| Age (years) | 1.1 (1.01-1.2) | **0.04** |
| FPG at FPV (mg/dL) | 1.3 (1.2-1.3) | **<0.001** |
| HbA1c (%) | 2.1 (0.6-6.8) | 0.24 |
| Family history of DM | 1.2 (0.6-2.3) | 0.61 |
| Pre-pregnancy BMI (kg/m2) | 1.0 (0.9-1.1) | 0.78 |
| GWG at 24-28 gestational weeks (kg) | 1.1 (0.9-1.2) | 0.17 |

BMI, body mass index; DM diabetes mellitus; FPG, fasting plasma glucose; FPV, first prenatal visit; GDM, gestational diabetes mellitus; GWG, gestational weight gain; HbA1c, hemoglobin A1c

In Result (page 10-11, line 155-169): A total of 1065 women who had their FPV between January 2013 and June 2018 were enrolled consecutively in the training cohort and another 182 women were included in the validation cohort from January 2019 to December 2019. The standard procedures in these cohorts were as follows. 1. FPG and HbA1c were measured at FPV to diagnose pre-existing diabetes (defined as FPG ≥126 mg/dL or HbA1c ≥ 6.5% at FPV) and GDM (FPG at FPV≥92 mg/dl). 2. In women without pre-existing diabetes and GDM, OGTT was performed at 24-28 gestational weeks. Of the 1065 women who agreed to join our research, 55 women were excluded because of twin pregnancies, history of GDM, or pre-existing diabetes. We also excluded 419 women who were recruited at the second trimester and their FPG at the FPV could not be obtained. Another 79 women were excluded because they received OGTT in other clinic or hospital and the data were not available. As a result, 512 women were included in our study. A flowchart of the study population is shown in Figure 1. Besides, another 182 women were included from January 2019 to December 2019 as the validation cohort. All the study subjects in the training and validation cohorts were Chinese Han.

In Result (page 12-13, line 184-191):

Table 1. Clinical characteristics and laboratory test results at the first prenatal visit and OGTT result at 24-28 gestational weeks in pregnancy women with and without gestational diabetes mellitus (GDM) in the training cohort and validation cohort.

|  | The training cohort | | | The validation cohort | | |
| --- | --- | --- | --- | --- | --- | --- |
| Baseline characteristics | Non-GDM | GDM | P value | Non-GDM | GDM | P value |
| N | 437 (85.4 %) | 75 (14.6 %) | NA | 125 (80.2 %) | 26 (17.2 %) | NA |
| Age (years) | 33.5 (4.1) | 35.1 (4.1) | **0.002** | 33.3 (3.6) | 33.8 (2.9) | 0.55 |
| Age≥35 (N, %) | 190 (43.5 %) | 42 (56 %) | **0.04** | 46 (36.8 %) | 11 (42.3 %) | 0.32 |
| Nulliparous (N, %) | 142 (32.5 %) | 26 (34.7 %) | 0.68 | 46 (36.8 %) | 13 (50 %) | 0.48 |
| Gestational age at the FPV (weeks) | 10.1 (1.9) | 10.3 (2.2) | 0.81 | 10 (1.5) | 9.9 (1.5) | 0.58 |
| Family history of DM (N, %) | 96 (22 %) | 26 (34.7 %) | **0.02** | 21 (16.8 %) | 5 (19.2 %) | 0.81 |
| History of PCOS | 16 (3.7 %) | 3 (4 %) | 0.89 | 7 (5.6 %) | 3 (11.5 %) | 0.27 |
| History of macrosomia | 2 (0.5 %) | 2 (2.7 %) | 0.05 | 0 | 0 | - |
| Pre-pregnancy BW (kg) | 55.3 (8.4) | 57.8 (14) | **0.03** | 55.2 (8.1) | 60.2 (8.5) | **0.005** |
| Pre-pregnancy BMI (kg/m2) | 21.9 (3.3) | 22.9 (5.3) | **0.02** | 21.9 (3.2) | 23.7 (3.5) | **0.02** |
| **GWG at 24-28 gestational weeks (kg)** | **6.3 (3.7)** | **6.3 (2.7)** | **0.4** | **6.1 (3.2)** | **6.2 (2.3)** | **0.92** |
| Laboratory test results at the first prenatal visit | | | |  |  |  |
| FPG (mg/dL) | 81.1 (5) | 88 (7.4) | **<0.001** | 82.8 (4.7) | 90.9 (7.2) | **<0.001** |
| HbA1c (%) | 5.2 (0.2) | 5.4 (0.3) | **<0.001** | 5.1 (0.3) | 5.3 (0.3) | **<0.001** |
| Glucose level during OGTT at 24-28 gestational weeks | | | |  |  |  |
| FPG during OGTT (mg/dL) | 77.9 (5) | 82.3 (6.9) | **<0.001** | 78.8 (5) | 85.2 (5.4) | **<0.001** |
| 1hPG (mg/dL) | 128.2 (24.3) | 164.7 (29.5) | **<0.001** | 127.6 (27.3) | 149 (34.3) | **<0.001** |
| 2hPG (mg/dL) | 109.9 (19.9) | 150.2 (29.4) | **<0.001** | 108.6 (21.3) | 135.6 (39.7) | **<0.001** |

Mean (standard deviations) or N (%) were shown. BMI, body mass index; BW, body weight; DM, diabetes mellitus; FPG, fasting plasma glucose; FPV, first prenatal visit; GDM, gestational diabetes mellitus; GWG, gestational weight gain; HbA1c, hemoglobin A1c; OGTT, oral glucose tolerance tests; FPG during OGTT, fasting plasma glucose during oral glucose tolerance tests; PCOS, Polycystic ovary syndrome; 1hPG, 1-hour plasma glucose during oral glucose tolerance tests; 2hPG, 2-hour plasma glucose during oral glucose tolerance tests

In S2 Table:

S2 Table. The relationship between clinical characteristics at the first prenatal visit and of gestational diabetes mellitus (GDM) at early pregnancy. Adjusted odds ratios (OR), 95% confidence intervals (95% CI) and p values in multivariate logistic regression models are shown. GDM was used as the dependent variable, and age, fasting plasma glucose, HbA1c, Family history of DM, and Pre-pregnancy BMI were used as the independent variables.

|  | OR (95% CI) | p |
| --- | --- | --- |
| Age (years) | 1.1 (1.01-1.2) | **0.03** |
| FPG at FPV (mg/dL) | 1.2 (1.2-1.3) | **<0.001** |
| HbA1c (%) | 2.3 (0.7-6.8) | 0.16 |
| Family history of DM | 1.2 (0.6-2.3) | 0.49 |
| Pre-pregnancy BMI (kg/m2) | 1.0 (0.9-1.1) | 0.81 |

BMI, body mass index; BW, body weight; DM diabetes mellitus; FPG, fasting plasma glucose; FPV, first prenatal visit; GDM, gestational diabetes mellitus; HbA1c, hemoglobin A1c

13. It is not personally clear whether a univariate or multivariate analysis was performed. These are very important data for your work and I would try to express them more clearly.

*Ans*: The analysis was multivariate. We have revised the manuscript to make it clearer.

In S2 Table: Adjusted odds ratios (OR), 95% confidence intervals (95% CI) and p values in multivariate logistic regression models are shown. GDM was used as the dependent variable, and age, fasting plasma glucose, HbA1c, family history of DM, and pre-pregnancy BMI were used as the independent variables.

14. “Such studies have shown that plasma glucose levels progressively increase with age.” Please report references.

*Ans*: Thanks for your suggestion. We have revised the manuscript and added the reference according to your suggestions.

In Discussion (page 23, line 278-279): In non-pregnant adults, plasma glucose levels increase with age (16).

15. “Our study demonstrates that GDM can be accurately predicted in early pregnancy based on simple maternal clinical parameters available at the time of the first visit blood screening.” The hypothesis of your study is that a first trimester screening for gestational diabetes based on risk factors could be performed. This statement on a study carried out on 547 women, of whom only 98 diagnosed with gestational diabetes, can accurately predict gestational diabetes, seems very strong to me.

*Ans*: Thanks for your comment. We have revised the wording according to your suggestion.

In Discussion (page 24, line 303-304): Our study suggests a potential way to predict GDM at the time of the first prenatal visit.

16. However, the limitations of this study is that all the study subjects were Han Chinese, studies on other ethnic groups should be done to see if the findings could be generalized. Are there no weaknesses to the study? Please add.

*Ans*: We have added weakness of the study according to your suggestion.

In Discussion (page 26, line 337-342): However, the study has several limitations. Firstly, all the study subjects were Han Chinese. Studies on other ethnic groups should be done to see if the findings could be generalized. Secondly, gestational weight gain (GWG) at the FPV was not recorded. Therefore, we could not know if GWG at the FPV could improve the performance of the algorithm or not. Thirdly, the sample size is relatively small. Further studies with larger sample size are needed to verify the findings in this study.

17. “In conclusion, FPG at the FPV and the prevalence of GDM increase by age. The screening algorithm using maternal age plus FPG at the FPV can greatly simplify the IADPSG diagnostic algorithm and reduce the use of the OGTT, especially in populations with a significant proportion of women who become pregnant at older ages. The optimal cutoff value for age plus FPG at the FPV is 115.” Sea above, this conclusion is too strong for such a reduced study cohort. Furthermore, in literature other methods are described to implement diabetes screening already in the first trimester as algorithms, maternal characteristics, ultrasound evaluation of maternal adipose tissue, etc… personally I would quote some.

*Ans*: Thanks for your suggestion. We have revised the manuscript according to your suggestions as below.

In Discussion (page 26-27, line 343-349): In conclusion, FPG at the FPV and the prevalence of GDM increase by age. By using maternal age plus FPG at the FPV, we suggest a way to diagnose GDM with reduced number of people taking OGTT. This may be especially useful in populations with a significant proportion of women getting pregnant until older ages. Our results provide an alternative screening strategy to previously published algorithms using the combination of various risk factors of GDM, such as history of GDM, family history of diabetes, ethnicity, parity, and BMI **(30, 31).**

Manuscript No.: PONE-D-20-00081

Title: Simplifying the gestational diabetes screen by maternal age plus fasting plasma glucose at first prenatal visit: a prospective cohort study

PLOS ONE

Responses to Reviewer #2:

Thank you very much for your constructive suggestions for this manuscript. We have made the revisions according to the comments, point by point, as follows. The page and line are according to revised manuscript with track changes.

1. I don’t understand the subtitle: “Early screen of gestational diabetes: role of maternal age” appears to be redundant or inserted by mistake.

*Ans*: We have revised the subtitle as below.

**“Short title: Early screening strategy of gestational diabetes”**

**Abstract**

2. As below – FPG at FPV is not universally performed or recommended

*Ans*: Thanks for your suggestion. We have revised the manuscript according to your suggestions as below.

In Abstract (page 3, line 35-37): Since FPG at the first prenatal visit (FPV) is a screening option for pre-existing diabetes, we evaluated the performance of age plus FPG at the FPV to reduce the need for the OGTT.

3. 547 were not consecutively recruited as > 1000 were eligible and approached

*Ans*: We have rephrased the sentence according to your question.

In Abstract (page 3, line 38-41): Pregnant women were recruited consecutively in 2013-2018 (the training cohort) and 2019 (the validation cohort). We excluded women with twin pregnancies, unavailable FPG at the FPV or OGTT data, pre-pregnancy diabetes, or a history of GDM.

**Introduction**

4. line 54-57: Inaccurate - ADA has allowed the OPTION of one step 75gram OGTT but also allow the two step 50 g GCT/100gram GTT

*Ans*: Thanks for your suggestion. We have revised the manuscript according to your suggestions as below.

In Introduction (page 5, line 59-64): For the diagnosis of gestational diabetes mellitus (GDM), the International Association of Diabetes and Pregnancy Study groups (IADPSG) and the World Health Organization (WHO) recommends the use of 75-g oral glucose tolerance test (OGTT) for all pregnant women at 24–28 weeks of gestation, which is also a diagnostic option suggested by the American Diabetes Association (ADA) in addition to the two-step method **(1-3).**

5. FPG at first visit is NOT universally recommended by ADA – only in those with risk factors. Even in those the recommendation is to test using “standard criteria”

*Ans*: Thanks for your suggestion. We have revised the manuscript according to your suggestions as below.

In Introduction (page 5-6, line 72-76): On the other hand, the IADPSG recommends the measurement of FPG during early pregnancy to detect pre-existing diabetes and GDM **(1, 9)**; whereas the ADA suggests the screening of undiagnosed type 2 diabetes in the first prenatal visit (FPV) in women with risk factors by the standard criteria, such as FPG, hemoglobin A1c or even OGTT **(3).**

6. Line 64 – 66 again, FPG not universally performed at FPV or first trimester (likely the same)

*Ans*: Thanks for your suggestion. We have revised the manuscript according to your suggestions as below.

In Introduction (page 5-6, line 72-76): On the other hand, the IADPSG recommends the measurement of FPG during early pregnancy to detect pre-existing diabetes and GDM (1, 9); whereas the ADA suggests the screening of undiagnosed type 2 diabetes in the first prenatal visit (FPV) in women with risk factors by the standard criteria, such as FPG, hemoglobin A1c or even OGTT **(3).**

7. Line 67-69 – sentence contradicts itself if “poorly predictive” of GDM in early pregnancy why would FPG at the FPV (which is also early pregnancy) be a “good predictor” ?

*Ans*: Thanks for your suggestion. We have corrected these sentences.

In Introduction (page 6, line 76-79): Since a higher first-trimester FPG level is associated with an increased risk of GDM diagnosed at 24-28 weeks (10), the FPG at the FPV may be a good predictor to be used in the screening of GDM.

8. Line 70: Really? Age is more independently important than obesity, family history previous GDM etc?

*Ans*: Thanks for your suggestion. We have revised the wording of the sentence.

In Introduction (page 6, line 80-84): Although increasing maternal age is known to be a significant risk factor of diabetes mellitus (DM) (11), information regarding the relationship between age and GDM is reported relatively rarely. In most developed countries, women get pregnant at older ages (12-15). Therefore, maternal age may be another important factor to be considered in screening of GDM, especially in countries where women become pregnant at an older age.

**Methods**

9. If I understand correctly, women were recruited at FPV and were eligible if they were singleton, viable and had no known preexisting DM. Then they had FPG and A1C. If they had PDM after these tests they were excluded. All that remained had a OGTT at 24-28 weeks. This should be clarified and shown in a flow diagram with numbers as Fig 1 (not as supplementary material)

*Ans*: Thanks for your suggestion. We have revised the manuscript according to your suggestions. The S1 figure was re-numbered to Fig 1 in the revised version.

In Result (page 10-11, line 155-167): A total of 1065 women who had their FPV between January 2013 and June 2018 were enrolled consecutively in the training cohort and another 182 women were included in the validation cohort from January 2019 to December 2019. The standard procedures in these cohorts were as follows. 1. FPG and HbA1c were measured at FPV to diagnose pre-existing diabetes (defined as FPG ≥126 mg/dL or HbA1c ≥ 6.5% at FPV) and GDM (FPG at FPV≥92 mg/dl). 2. In women without pre-existing diabetes and GDM, OGTT was performed at 24-28 gestational weeks. Of the 1065 women who agreed to join our research, 55 women were excluded because of twin pregnancies, history of GDM, or pre-existing diabetes. We also excluded 419 women who were recruited at the second trimester and their FPG at the FPV could not be obtained. Another 79 women were excluded because they received OGTT in other clinic or hospital and the data were not available. As a result, 512 women were included in our study. A flowchart of the study population is shown in Figure 1.

Figure 1. The flow chart for the inclusion and exclusion of the study population.

10. From figure S1 – I note that nearly half of the base population did not have FPG and A1C. In line 92 you say that “ALL pregnant women were done with FPG and hemoglobin A1c (HbA1c) …” Please clarify this. Was in fact FPG and A1C a standardized protocol at the time of the study? If so why did > 400 women not have this done. Were they perhaps different clinically than those that did? Were all 1047 approached and consented if singleton and no PDM? If so – did the 419 without FPG and A1C not consent? This is all confusing and needs to be clarified.

*Ans*: Thanks for your suggestion. The standard procedure in this study was to measure FPG and HbA1c at FPV. In women without pre-existing diabetes and GDM diagnosed by FPG at FPV, OGTT was performed at 24-28 weeks. Since some women visited our hospital later and were recruited in the second trimester, their FPG and HbA1c at FPV were not available and were excluded from the analysis. We have revised our manuscript and described the standard procedure. Besides, we have compared women with and without data of FPG and HbA1c at FPV. As sown in S1 Table, there was no significant difference in clinical characteristics between these two groups of women. We have added these results in Results and S1 Table.

In Result (page 10-11, line 155-167): A total of 1065 women who had their FPV between January 2013 and June 2018 were enrolled consecutively in the training cohort and another 182 women were included in the validation cohort from January 2019 to December 2019. The standard procedures in these cohorts were as follows. 1. FPG and HbA1c were measured at FPV to diagnose pre-existing diabetes (defined as FPG ≥126 mg/dL or HbA1c ≥ 6.5% at FPV) and GDM (FPG at FPV≥92 mg/dl). 2. In women without pre-existing diabetes and GDM, OGTT was performed at 24-28 gestational weeks. Of the 1065 women who agreed to join our research, 55 women were excluded because of twin pregnancies, history of GDM, or pre-existing diabetes. We also excluded 419 women who were recruited at the second trimester and their FPG at the FPV could not be obtained. Another 79 women were excluded because they received OGTT in other clinic or hospital and the data were not available. As a result, 512 women were included in our study. A flowchart of the study population is shown in Figure 1.

Figure 1. The flow chart for the inclusion and exclusion of the study population.

In Results (page 11, line 169-172): In S1 Table, clinical characteristics between the 512 women with FPG and HbA1c at FPV and the 419 women without FPG and HbA1c at FPV were compared. There was no significant difference in clinical characteristics between these two groups.

S1 table. Clinical characteristics and laboratory test results at the first prenatal visit and OGTT result at 24-28 gestational weeks in pregnancy women with and without FPG and HbA1c at the first prenatal visit.

| Baseline characteristics | **With** FPG and HbA1c at the FPV | **Without** FPG and HbA1c at the FPV | P value |
| --- | --- | --- | --- |
| N | 512 | 407 |  |
| Age (years) | 33.8 (4.2) | 34 (4) | 0.36 |
| Age≥35 (N, %) | 232 (45.3 %) | 180 (44.2 %) | 0.63 |
| Nulliparous (N, %) | 168 (32.8 %) | 163 (40 %) | 0.68 |
| Gestational age at the FPV (weeks) | 10.1 (2) | 10.9 (2.6) | 0.81 |
| Family history of DM (N, %) | 122 (23.8 %) | 79 (19.4 %) | 0.11 |
| History of PCOS | 19 (3.7 %) | 14 (3.4 %) | 0.83 |
| History of macrosomia | 4 (0.8 %) | 2 (0.5 %) | 0.59 |
| Pre-pregnancy BW (kg) | 55.6 (9.5) | 56.2 (9.2) | 0.34 |
| Pre-pregnancy BMI (kg/m2) | 22 (3.7) | 22 (3.3) | 0.89 |
| GWG at 24-28 gestational weeks (kg) | 6.3 (3.5) | 6.7 (3.3) | 0.07 |
| Glucose level during OGTT at 24-28 gestational weeks | | | |
| FPG during OGTT (mg/dL) | 79.2 (7) | 78.6 (5.5) | 0.13 |
| 1hPG (mg/dL) | 133.9 (29) | 133.6 (28.2) | 0.86 |
| 2hPG (mg/dL) | 115.5 (24.9) | 115.8 (25.8) | 0.86 |

Mean (standard deviations) or N (%) were shown.

BMI, body mass index; BW, body weight; DM, diabetes mellitus; FPG, fasting plasma glucose; FPV, first prenatal visit; GDM, gestational diabetes mellitus; GWG, gestational weight gain; HbA1c, aemoglobin A1c; OGTT, oral glucose tolerance tests; FPG during OGTT, fasting plasma glucose during oral glucose tolerance tests; PCOS, Polycystic ovary syndrome; 1hPG, 1-hour plasma glucose during oral glucose tolerance tests; 2hPG, 2-hour plasma glucose during oral glucose tolerance tests

11. Was there a gestational age criteria for the FPV? If someone had a FPV at 20 weeks was she included? Please clarify

*Ans*: No, there was no gestational age criteria for inclusion. The obstetricians arranged FPG and HbA1c tests at FPV, which was usually in the first trimester. If the women participated the study later, such as in the second trimester, or if she had FPG and HbA1c tested in another hospital, FPG and HbA1c tests would not be repeated, which was judged by the obstetricians. The mean gestational age to measure FPG and HbA1c at FPV was 10 weeks in this study. We have revised the manuscript to make it clearer.

In Result (page 10-11, line 155-167): A total of 1065 women who had their FPV between January 2013 and June 2018 were enrolled consecutively in the training cohort and another 182 women were included in the validation cohort from January 2019 to December 2019. The standard procedures in these cohorts were as follows. 1. FPG and HbA1c were measured at FPV to diagnose pre-existing diabetes (defined as FPG ≥126 mg/dL or HbA1c ≥ 6.5% at FPV) and GDM (FPG at FPV≥92 mg/dl). 2. In women without pre-existing diabetes and GDM, OGTT was performed at 24-28 gestational weeks. Of the 1065 women who agreed to join our research, 55 women were excluded because of twin pregnancies, history of GDM, or pre-existing diabetes. We also excluded 419 women who were recruited at the second trimester and their FPG at the FPV could not be obtained. Another 79 women were excluded because they received OGTT in other clinic or hospital and the data were not available. As a result, 512 women were included in our study. A flowchart of the study population is shown in Figure 1.

12. When defining BMI was pre-pregnancy BMI used? Early pregnancy?

*Ans*: Thanks for your suggestion. The pre-pregnancy weight was acquired by questionnaire. We have revised the manuscript to make it clearer.

In Research Design and Methods (page 8, line 113-116): Their clinical characteristics were acquired by questionnaire and recorded such as age, parity, pre-pregnancy BMI, history of GDM and polycystic ovary syndrome (PCOS), family history of diabetes (defined as first degree relative with diabetes mellitus), etc**.**

13. Were the FPG and A1C results available to the clinicians? What was done if at FPV the FPG was > 5.1 or A1C as 5.9-6.4?

*Ans*: FPG and A1C results at FPV were available to the clinicians. Women with FPG at FPV ≥ 92 mg/dl and <126 mg/dl were diagnosed as gestational diabetes according to the IADPSG criteria. They did not receive OGTT in the second trimester. Women with HbA1c <6.5% and FPG<92 mg/dl at FPV were regarded as normal and would receive OGTT at 24-28 gestational weeks. In order to be clearer, we have revised the Research Design and Methods.

In Research Design and Methods (page 7-8, line 107-112): Eligible participants received blood tests for FPG and haemoglobin A1c (HbA1c) at the FPV after fasting for 8-10 hours. Women with FPG at the FPV less than 92 mg/dL received 75-g OGTT at 24-28 gestational weeks. The diagnosis of GDM was based on the recommendations of the IADPSG if FPG at the FPV ≥92 mg/dL, or if one of the results from the OGTT exceeded the cutoffs, including FPG ≥92 mg/dL, plasma glucose at 1h ≥180 mg/dL, or plasma glucose at 2h ≥153 mg/dL **(1).**

**Results**

14. Was gestational weight gain collected and if not – why?

*Ans*: Yes, we have collected gestational weight gain (GWG). These data have been added in Table 1.

In Result (page 12-13, line 184-191):

Table 1. Clinical characteristics and laboratory test results at the first prenatal visit and OGTT result at 24-28 gestational weeks in pregnancy women with and without gestational diabetes mellitus (GDM) in the training cohort and validation cohort.

|  | The training cohort | | | The validation cohort | | |
| --- | --- | --- | --- | --- | --- | --- |
| Baseline characteristics | Non-GDM | GDM | P value | Non-GDM | GDM | P value |
| N | 437 (85.4 %) | 75 (14.6 %) | NA | 125 (80.2 %) | 26 (17.2 %) | NA |
| Age (years) | 33.5 (4.1) | 35.1 (4.1) | **0.002** | 33.3 (3.6) | 33.8 (2.9) | 0.55 |
| Age≥35 (N, %) | 190 (43.5 %) | 42 (56 %) | **0.04** | 46 (36.8 %) | 11 (42.3 %) | 0.32 |
| Nulliparous (N, %) | 142 (32.5 %) | 26 (34.7 %) | 0.68 | 46 (36.8 %) | 13 (50 %) | 0.48 |
| Gestational age at the FPV (weeks) | 10.1 (1.9) | 10.3 (2.2) | 0.81 | 10 (1.5) | 9.9 (1.5) | 0.58 |
| Family history of DM (N, %) | 96 (22 %) | 26 (34.7 %) | **0.02** | 21 (16.8 %) | 5 (19.2 %) | 0.81 |
| History of PCOS | 16 (3.7 %) | 3 (4 %) | 0.89 | 7 (5.6 %) | 3 (11.5 %) | 0.27 |
| History of macrosomia | 2 (0.5 %) | 2 (2.7 %) | 0.05 | 0 | 0 | - |
| Pre-pregnancy BW (kg) | 55.3 (8.4) | 57.8 (14) | **0.03** | 55.2 (8.1) | 60.2 (8.5) | **0.005** |
| Pre-pregnancy BMI (kg/m2) | 21.9 (3.3) | 22.9 (5.3) | **0.02** | 21.9 (3.2) | 23.7 (3.5) | **0.02** |
| GWG at 24-28 gestational weeks (kg) | 6.3 (3.7) | 6.3 (2.7) | 0.4 | 6.1 (3.2) | 6.2 (2.3) | 0.92 |
| Laboratory test results at the first prenatal visit | | | |  |  |  |
| FPG (mg/dL) | 81.1 (5) | 88 (7.4) | **<0.001** | 82.8 (4.7) | 90.9 (7.2) | **<0.001** |
| HbA1c (%) | 5.2 (0.2) | 5.4 (0.3) | **<0.001** | 5.1 (0.3) | 5.3 (0.3) | **<0.001** |
| Glucose level during OGTT at 24-28 gestational weeks | | | |  |  |  |
| FPG during OGTT (mg/dL) | 77.9 (5) | 82.3 (6.9) | **<0.001** | 78.8 (5) | 85.2 (5.4) | **<0.001** |
| 1hPG (mg/dL) | 128.2 (24.3) | 164.7 (29.5) | **<0.001** | 127.6 (27.3) | 149 (34.3) | **<0.001** |
| 2hPG (mg/dL) | 109.9 (19.9) | 150.2 (29.4) | **<0.001** | 108.6 (21.3) | 135.6 (39.7) | **<0.001** |

Mean (standard deviations) or N (%) were shown. BMI, body mass index; BW, body weight; DM, diabetes mellitus; FPG, fasting plasma glucose; FPV, first prenatal visit; GDM, gestational diabetes mellitus; GWG, gestational weight gain; HbA1c, hemoglobin A1c; OGTT, oral glucose tolerance tests; FPG during OGTT, fasting plasma glucose during oral glucose tolerance tests; PCOS, Polycystic ovary syndrome; 1hPG, 1-hour plasma glucose during oral glucose tolerance tests; 2hPG, 2-hour plasma glucose during oral glucose tolerance tests

15. Why is FPG of >91 at FPV (10 weeks mean) diagnostic of GDM (Fig 2)?? This is not standard. The IADPSG values are extrapolated from HAPO which used GTT values at 24-28 weeks. There is no outcome based validated thresholds for early GTT. Were these women treated (see comment 11)

*Ans*: We have re-checked the diagnostic criteria by the IADPSG and confirmed that FPG value in early pregnancy ≥92 mg/dl and <126 mg/dl is a diagnostic criterion of GDM by the IADPSG (Diabetes Care. 2010;33(3):676-82.). In this study, these women with FPG at FPV ≥92 mg/dl and <126 mg/dl were treated and did not receive 75-g OGTT in the second trimester.

16. Line 142-143: In addition to age, previous GDM was also independently associated with GDM OR 8.14. This is not mentioned. Also what about the effect of BMI? It was significant on the univariate analysis and it is mentioned in the legend (and in line 232 of the discussion) but not in the body of the table. What happened to this variable?

*Ans*: Thanks for your valuable opinion. We agree with you that previous GDM is an important risk factor of GDM. In clinical practice, women with previous GDM usually receive OGTT and do not need an algorithm to decide if OGTT should be done. Therefore, we have excluded these women and re-performed the analyses. The findings were similar. We have revised the manuscript for these changes. As for the effect of BMI, we have added it in the multivariate analysis and revised the table (S2 Table).

In Methods (page 7, line 104-107): Since it is reasonable for pregnant women with a history of GDM to undergo OGTT, we excluded women with a history of GDM from the analyses in this study (women with history of GDM, N=35 in the training cohort, and N=7 in the validation cohort).

In Result (page 14, line 201-203): Age and FPG at the FPV were the independent predictors of GDM in multivariate logistic model (S2 Table). Since the odds ratios and regression coefficients for age and FPG were similar, "age plus the FPG" was used to develop a screening algorithm for GDM.

S2 Table. The relationship between clinical characteristics at the first prenatal visit and of gestational diabetes mellitus (GDM) at early pregnancy. Adjusted odds ratios (OR), 95% confidence intervals (95% CI) and p values in multivariate logistic regression models are shown. GDM was used as the dependent variable, and age, fasting plasma glucose, HbA1c, family history of DM, and pre-pregnancy BMI were used as the independent variables.

|  | OR (95% CI) | p |
| --- | --- | --- |
| Age (years) | 1.1 (1.01-1.2) | **0.03** |
| FPG at FPV (mg/dL) | 1.2 (1.2-1.3) | **<0.001** |
| HbA1c (%) | 2.3 (0.7-6.8) | 0.16 |
| Family history of DM | 1.2 (0.6-2.3) | 0.49 |
| Pre-pregnancy BMI (kg/m2) | 1.0 (0.9-1.1) | 0.81 |

BMI, body mass index; BW, body weight; DM diabetes mellitus; FPG, fasting plasma glucose; FPV, first prenatal visit; HbA1c, hemoglobin A1c

**Discussion**

17. Discussion overall VERY repetitive.

*Ans*: Thanks for your suggestion. We have extensively revised the Discussion section and deleted the repetitive parts. Besides, we have sent the manuscript for English language editing. We think the Discussion section is more concise in the revised version.

18. Line 227 – provide references for evidence that early identification of “GDM” leads to better outcomes

*Ans*: We agree with your that there is no evidence showing that early identification of GDM leads to better outcomes in the literature. Besides, the aim of the study is to reduce the OGTT needed to diagnose GDM, but not to diagnose GDM earlier. Therefore, we have deleted the sentences.

19. These results are not consistent with the study by Zhu et al DIABETES CARE, VOLUME 36, MARCH 2013. In this study even if FPG at FPV as < 4.1 8.1% had a subsequent diagnosis of GDM at 24-28 weeks. In addition, they were able to show that overall – there was poor correlation between the FPG at FPV and the FPG at the OGTT. 100% specificity was achieved only > 5.7 mmol (100.8) and with corresponding low sensitivity. Please explain the difference between this (large) study’s results and yours. In addition, discuss why FPG performs favorably as a screening tool in this study in contrast with what is known regarding the poor performance of early FPG as a screening tool for future GDM (See summary of evidence in NICE guidelines).

*Ans*: In the study by Zhu et al., 8.1% of women with FPG at FPV<4.1 mmol/l had a subsequent diagnosis of GDM. These women were false negatives by FPG at FPV, and false negatives will reduce the sensitivity, but not the specificity. On the other hand, specificity equals to 1 – false positive rate. In our study, pregnant women were diagnosed as GDM either by FPG at the FPV≥92 mg/dL or the results of OGTT in both the "FPG at FPV" algorithm and the "age plus FPG at FPV" algorithm. Therefore, the false positive rate was 0% and the specificity was 100% for both algorithms, no matter which cutoffs were chosen. However, in the study by Zhu et al., they used different diagnostic criteria for GDM, and FPG at the FPV ≥92 mg/dl was not one of the diagnostic criteria. As a result, the specificity varied by cutoffs of FPG. Besides, in the study by Zhu et al., they showed that FPG at the FPV could only identify 8.8% of women with FPG>5.1 mmol/l (92 mg/dl). This is not the goal of our study. The aim of our study is to use FPG at the FPV with/without age to reduce OGTT%. We have revised the manuscript to discuss the differences between Zhu’s study and our study.

In the NICE guidelines, the rationales or supporting evidence for not recommending FPG as a screening tool are not mentioned in the texts nor the summary of evidence. Therefore, we cannot comment on this. However, different diagnostic criteria for GDM were used in the NICE guideline, ie. fasting plasma glucose greater than 100 mg/dl or 2-hour plasma glucose greater than 140 mg/dl. This may contribute to the recommendation about FPG. We have revised the manuscript to discuss these differences.

In Discussion (page 24-25, line 305-326): However, there is another study which evaluated the performance of FPG at FPV to predict GDM, and performance was not as good as our results (27). There are some differences between that study and the present study. First, the diagnostic criteria of GDM were different. In their study, GDM was diagnosed when any one of the following value was met or exceeded during the 75g OGTT at 24-28 weeks, including fasting plasma glucose ≥92 mg/dL, 1-hour plasma glucose ≥180 mg/dL or 2-hour plasma glucose, ≥153 mg/dL. FPG at FPV ≥92 mg/dL was not a criterion to diagnose GDM. In our study, GDM was diagnosed according to the IADPSG criteria, which means that women with FPG at FPV ≥92 mg/dL, or data from OGTT exceeds the above-mentioned cutoffs were diagnosed GDM. As a result, the false positive rate was 0% in our algorithm, no matter which cutoffs were chosen. By definition, specificity equals to 1 – false positive rate. Therefore, the specificity in the algorithm is always 100%. In addition, although the mean age of the cohort in that paper was not described, we believe that it would not be high, since women often get pregnant at relatively young ages in China. In contrast, the mean age in our cohort was 33.7 years. All these differences may lead to the different results in these two studies. In addition, the NICE guidelines do not recommend the use of FPG to assess risk of developing GDM (29). However, the NICE guidelines suggest a different criteria to diagnose GDM, ie. fasting plasma glucose greater than 100 mg/dl or 2-hour plasma glucose greater than 140 mg/dl. Therefore, the difference in the diagnostic criteria may contribute to the discrepancies between the recommendation from the NICE guidelines and the present study.

20. Missing references for other predictive models including recent: Zheng, T., Ye, W., Wang, X. et al. A simple model to predict risk of gestational diabetes mellitus from 8 to 20 weeks of gestation in Chinese women. BMC Pregnancy Childbirth 19, 252 (2019). <https://doi.org/10.1186/s12884-019-2374-8>.

*Ans*: We have added the reference according to your suggestions.

In Discussion (page 24, line 303-305): Our study suggests a potential way to predict GDM at the time of the first prenatal visit. We found that age and FPG at the FPV were the independent predictors for the development of GDM, which was supported by a report in the literature **(28)**.

21. Missing many of the other limitations:
a. The “missing” 419 women that did not have FPG and A1C at FPV. If you can show that their characteristics are not different from those that had testing then that will provide some support for the validity of the results

*Ans*: Thanks for your suggestion. We have performed the analysis. The clinical characteristics between these women and the women included in the study were similar. We have added the information in S1 table.

In Result (page 11, line 169-172): In S1 Table, clinical characteristics between the 512 women with FPG and HbA1c at FPV and the 419 women without FPG and HbA1c at FPV were compared. There was no significant difference in clinical characteristics between these two groups.

S1 table. Clinical characteristics and laboratory test results at the first prenatal visit and OGTT result at 24-28 gestational weeks in pregnancy women with and without FPG and HbA1c at the first prenatal visit.

| Baseline characteristics | **With** FPG and HbA1c at the FPV | **Without** FPG and HbA1c at the FPV | P value |
| --- | --- | --- | --- |
| N | 512 | 407 |  |
| Age (years) | 33.8 (4.2) | 34 (4) | 0.36 |
| Age≥35 (N, %) | 232 (45.3 %) | 180 (44.2 %) | 0.63 |
| Nulliparous (N, %) | 168 (32.8 %) | 163 (40 %) | 0.68 |
| Gestational age at first recruitment (weeks) | 10.1 (2) | 26.5 (1.6) | - |
| Family history of DM (N, %) | 122 (23.8 %) | 79 (19.4 %) | 0.11 |
| History of PCOS | 19 (3.7 %) | 14 (3.4 %) | 0.83 |
| History of macrosomia | 4 (0.8 %) | 2 (0.5 %) | 0.59 |
| Pre-pregnancy BW (kg) | 55.6 (9.5) | 56.2 (9.2) | 0.34 |
| Pre-pregnancy BMI (kg/m2) | 22 (3.7) | 22 (3.3) | 0.89 |
| GWG at 24-28 gestational weeks (kg) | 6.3 (3.5) | 6.7 (3.3) | 0.07 |
| Glucose level during OGTT at 24-28 gestational weeks | | | |
| FPG during OGTT (mg/dL) | 79.2 (7) | 78.6 (5.5) | 0.13 |
| 1hPG (mg/dL) | 133.9 (29) | 133.6 (28.2) | 0.86 |
| 2hPG (mg/dL) | 115.5 (24.9) | 115.8 (25.8) | 0.86 |

Mean (standard deviations) or N (%) were shown.

BMI, body mass index; BW, body weight; DM, diabetes mellitus; FPG, fasting plasma glucose; FPV, first prenatal visit; GDM, gestational diabetes mellitus; GWG, gestational weight gain; HbA1c, hemoglobin A1c; OGTT, oral glucose tolerance tests; FPG during OGTT, fasting plasma glucose during oral glucose tolerance tests; PCOS, Polycystic ovary syndrome; 1hPG, 1-hour plasma glucose during oral glucose tolerance tests; 2hPG, 2-hour plasma glucose during oral glucose tolerance tests

b. The lack of clinician blinding

*Ans*: In this prospective study, data were analyzed after the recruitment. The clinicians used standard protocol to diagnose GDM and did not know the results of the analysis, such as the cutoff value chosen. We have described these details in Methods to make it clearer.

In Methods (page 7-8, line 107-112): Eligible participants received blood tests for FPG and haemoglobin A1c (HbA1c) at the FPV after fasting for 8-10 hours. Women with FPG at the FPV less than 92 mg/dL received 75-g OGTT at 24-28 gestational weeks. The diagnosis of GDM was based on the recommendations of the IADPSG if FPG at the FPV ≥92 mg/dL, or if one of the results from the OGTT exceeded the cutoffs, including FPG ≥92 mg/dL, plasma glucose at 1h ≥180 mg/dL, or plasma glucose at 2h ≥153 mg/dL (9). The clinicians were blinded for the results of the statistical analyses.

c. Unlike the authors previous study – there is no validation cohort

*Ans*: Thanks for your suggestion. We have added data from a validation cohort. Findings from the validation cohort support the results from the training cohort. We have revised the manuscript to report these findings.

In Research design and methods (page 7, line 94-99): Two cohort studies were conducted for this project, including a prospective cohort study to develop the screening algorithms (the training cohort) and the other cohort study to validate the performance of the algorithms (the validation cohort). The training cohort study was conducted between January 2013 and June 2018 which recruited pregnant women who visited the obstetric clinic of National Taiwan University Hospital. The pregnant women in the validation cohort was recruited in 2019.

In Result (page 16, line 234-236): Similarly, the OGTT% was decreased to 73.6% by the “FPG at the FPV” algorithm and 61.5% by the “age plus FPG at the FPV” algorithm in the validation cohort (Table 3).

In Result (page 18, line 245-249):

Table 3. Performance of “FPG at the FPV” algorithm and “age plus FPG at the FPV” algorithm to screen gestational diabetes mellitus (GDM) in the training cohort and in the validation cohort.

| Algorithm | Threshold to exclude GDM | Training cohort | | | Validation cohort | | |
| --- | --- | --- | --- | --- | --- | --- | --- |
|  |  | OGTT (%) | Sensitivity (%) | Specificity (%) | OGTT (%) | Sensitivity (%) | Specificity (%) |
| FPG at the FPV | < 79 | 68.8 | 92 (83.4-97) | 100 | 73.6 | 89 (73.9-96.9) | 100 |
| “Age plus FPG at the FPV” | < 114 | 58.5 | 90.7 (81.7-96.2) | 100 | 61.5 | 91.7 (77.5-98.2) | 100 |

Estimates (95% confidence interval) were shown.

FPG, fasting plasma glucose; FPV, first prenatal visit; GDM, gestational diabetes mellitus; OGTT, oral glucose tolerance tests;

FPG is in conventional units (mg/dL).

d. No reporting of gestational weight gain

*Ans*: We have added gestational weight gain (GWG) in Table 1 according to your suggestion.

In Result (page 12-13, line 184-191):

Table 1. Clinical characteristics and laboratory test results at the first prenatal visit and OGTT result at 24-28 gestational weeks in pregnancy women with and without gestational diabetes mellitus (GDM) in the training cohort and validation cohort.

|  | The training cohort | | | The validation cohort | | |
| --- | --- | --- | --- | --- | --- | --- |
| Baseline characteristics | Non-GDM | GDM | P value | Non-GDM | GDM | P value |
| N | 437 (85.4 %) | 75 (14.6 %) | NA | 125 (80.2 %) | 26 (17.2 %) | NA |
| Age (years) | 33.5 (4.1) | 35.1 (4.1) | **0.002** | 33.3 (3.6) | 33.8 (2.9) | 0.55 |
| Age≥35 (N, %) | 190 (43.5 %) | 42 (56 %) | **0.04** | 46 (36.8 %) | 11 (42.3 %) | 0.32 |
| Nulliparous (N, %) | 142 (32.5 %) | 26 (34.7 %) | 0.68 | 46 (36.8 %) | 13 (50 %) | 0.48 |
| Gestational age at the FPV (weeks) | 10.1 (1.9) | 10.3 (2.2) | 0.81 | 10 (1.5) | 9.9 (1.5) | 0.58 |
| Family history of DM (N, %) | 96 (22 %) | 26 (34.7 %) | **0.02** | 21 (16.8 %) | 5 (19.2 %) | 0.81 |
| History of PCOS | 16 (3.7 %) | 3 (4 %) | 0.89 | 7 (5.6 %) | 3 (11.5 %) | 0.27 |
| History of macrosomia | 2 (0.5 %) | 2 (2.7 %) | 0.05 | 0 | 0 | - |
| Pre-pregnancy BW (kg) | 55.3 (8.4) | 57.8 (14) | **0.03** | 55.2 (8.1) | 60.2 (8.5) | **0.005** |
| Pre-pregnancy BMI (kg/m2) | 21.9 (3.3) | 22.9 (5.3) | **0.02** | 21.9 (3.2) | 23.7 (3.5) | **0.02** |
| GWG at 24-28 gestational weeks (kg) | 6.3 (3.7) | 6.3 (2.7) | 0.4 | 6.1 (3.2) | 6.2 (2.3) | 0.92 |
| Laboratory test results at the first prenatal visit | | | |  |  |  |
| FPG (mg/dL) | 81.1 (5) | 88 (7.4) | **<0.001** | 82.8 (4.7) | 90.9 (7.2) | **<0.001** |
| HbA1c (%) | 5.2 (0.2) | 5.4 (0.3) | **<0.001** | 5.1 (0.3) | 5.3 (0.3) | **<0.001** |
| Glucose level during OGTT at 24-28 gestational weeks | | | |  |  |  |
| FPG during OGTT (mg/dL) | 77.9 (5) | 82.3 (6.9) | **<0.001** | 78.8 (5) | 85.2 (5.4) | **<0.001** |
| 1hPG (mg/dL) | 128.2 (24.3) | 164.7 (29.5) | **<0.001** | 127.6 (27.3) | 149 (34.3) | **<0.001** |
| 2hPG (mg/dL) | 109.9 (19.9) | 150.2 (29.4) | **<0.001** | 108.6 (21.3) | 135.6 (39.7) | **<0.001** |

Mean (standard deviations) or N (%) were shown. BMI, body mass index; BW, body weight; DM, diabetes mellitus; FPG, fasting plasma glucose; FPV, first prenatal visit; GDM, gestational diabetes mellitus; GWG, gestational weight gain; HbA1c, hemoglobin A1c; OGTT, oral glucose tolerance tests; FPG during OGTT, fasting plasma glucose during oral glucose tolerance tests; PCOS, Polycystic ovary syndrome; 1hPG, 1-hour plasma glucose during oral glucose tolerance tests; 2hPG, 2-hour plasma glucose during oral glucose tolerance tests

e. No reporting of perinatal outcomes. What are the outcomes for those missed by this strategy (90% sensitivity compared with 100% sensitivity with universal OGTT)

*Ans*: Thanks for your suggestion. We have added these data in Table 4 and S3 table.

In Result (page 19-20, line 261-270): The relationship between pregnancy outcomes and GDM defined by different algorithms was summarized in Table 4. The results showed that pregnant women with GDM, compared to those without GDM, had higher percentage of adverse maternal outcomes and fetal outcomes, no matter GDM was defined by “FPG at the FPV” algorithm, "age plus FPG at the FPV" algorithm or the IADPSG criteria (Table 4). Besides, there were 6 women with GDM missed by the “FPG at the FPV” algorithm (group 1) and 7 women with GDM missed by "age plus FPG at the FPV" algorithm (group 2). In S3 Table, there was no statistically significant difference between group 1 vs. group 3 and group 2 vs. group3, except that preterm delivery was more prevalent in group 1, compared with group 3.

In Result (page 21-22, line 271-276):

Table 4. Pregnancy outcomes in women with and without gestational diabetes mellitus defined by the FPG at FPV alone algorithm, the "age plus FPG at the FPV" algorithm, or the IADPSG criteria.

|  | FPG at FPV alone | | Age plus FPG at the FPV | | IADPSG | |
| --- | --- | --- | --- | --- | --- | --- |
|  | without GDM | with GDM | without GDM | with GDM | without GDM | with GDM |
| N | 426 | 63 | 427 | 62 | 420 | 69 |
| Maternal adverse outcomes | | | | | | |
| Primary CS (N, %) | 80 (18.8 %) | 13 (20.6 %) | 80 (18.7 %) | 13 (21 %) | 80 (19 %) | 13 (18.8 %) |
| PIH (N, %) | 6 (1.4 %) | 2 (3.2 %) | 6 (1.4 %) | 2 (3.2 %) | 6 (1.4 %) | 2 (2.9 %) |
| Preeclampsia (N, %) | 7 (1.6%) | 2 (3.2 %) | 7 (1.6%) | 2 (3.2 %) | 7 (1.7 %) | 2 (2.9 %) |
| Preterm delivery (N, %) | 21 (4.9 %) | 11 (17.5 %) * | 21 (4.9 %) | 11 (17.7 %) * | 19 (4.5 %) | 13 (18.8 %) * |
| Any adverse maternal outcome | 96 (22.5 %) | 22 (34.9 %) * | 95 (22.2 %) | 23 (37.2 %) * | 94 (22.4 %) | 24 (34.8 %) * |
| Fetal adverse outcomes | | | | | | |
| BW >90^th^ percentile (N, %) | 40 (9.4 %) | 8 (12.7 %) | 40 (9.4 %) | 8 (12.9 %) | 40 (9.3 %) | 8 (13 %) |
| Jaundice (N, %) | 69 (16.2 %) | 14 (22.2 %) | 68 (15.9 %) | 15 (24.2 %) | 67 (16 %) | 16 (23.2 %) |
| Admission to NICU (N, %) | 18 (4.2 %) | 3 (4.8 %) | 18 (4.2 %) | 3 (4.8 %) | 18 (4.3 %) | 3 (4.3 %) |
| Birth trauma (N, %) | 2 (0.5 %) | 0 (0 %) | 2 (0.5 %) | 0 (0 %) | 2 (0.5 %) | 0 (0 %) |
| Neonatal hypo-glycemia (N, %) | 6 (1.4 %) | 3 (4.8 %) | 6 (1.4 %) | 3 (4.8 %) | 6 (1.4 %) | 3 (4.3 %) |
| Fetal death (N, %) | 0 (0 %) | 0 (0 %) | 0 (0 %) | 0 (0 %) | 0 (0 %) | 0 (0 %) |
| Any adverse fetal outcome | 122 (28.6 %) | 26 (41.3 %) * | 123 (28.8 %) | 25 (40.3 %) * | 120 (28.6 %) | 28 (40.6 %) * |

*p<0.05 by chi-squared test, for subjects with or without GDM using different diagnostic algorithms

CS, Cesarean section; FPG, fasting plasma glucose; FPV, first prenatal visit; IADPSG, the International Association of the Diabetes and Pregnancy Study Group; PIH, pregnancy induced hypertension; BW >90^th^ percentile, a birth weight above the gender-specific 90^th^ percentile for gestational age; NICU, neonatal intensive care unit.

S3 Table. Pregnancy outcomes in women with false negative result defined by " FPG at the FPV" algorithm and "age plus FPG at the FPV" algorithm, comparing to the women with true negative result defined by the IADPSG criteria.

|  | Group 1  False negative by FPG at FPV alone | Group 2  False negative by age plus FPG at the FPV | Group 3  True negative by the IADPSG |
| --- | --- | --- | --- |
| N | 6 | 7 | 420 |
| Maternal adverse outcomes |  |  |  |
| Primary CS (N, %) | 0 (0 %) | 0 (0 %) | 80 (19 %) |
| PIH (N, %) | 0 (0 %) | 0 (0 %) | 6 (1.4 %) |
| Preeclampsia (N, %) | 0 (0 %) | 0 (0 %) | 7 (1.7 %) |
| Preterm delivery (N, %) | 2 (33.3 %) * | 1 (16.7 %) | 19 (4.5 %) |
| Any adverse maternal outcome | 2 (33.3 %) | 1 (16.7 %) | 94 (22.4 %) |
| Fetal adverse outcomes |  |  |  |
| BW >90th percentile (N, %) | 1 (16.7 %) | 1 (16.7 %) | 40 (9.3 %) |
| Jaundice (N, %) | 1 (16.7 %) | 2 (33.3 %) | 67 (16 %) |
| Admission to NICU (N, %) | 0 (0 %) | 0 (0 %) | 18 (4.3 %) |
| Birth trauma (N, %) | 0 (0 %) | 0 (0 %) | 2 (0.5 %) |
| Neonatal hypo-glycemia (N, %) | 0 (0 %) | 0 (0 %) | 6 (1.4 %) |
| Fetal death (N, %) | 0 (0 %) | 0 (0 %) | 0 (0 %) |
| Any adverse fetal outcome | 2 (33.3 %) | 3 (50 %) | 120 (28.6 %) |

* p<0.05 by Fisher’s exact test, for group 1 vs. group 3 and group 2 vs. group3

CS, Cesarean section; FPG, fasting plasma glucose; FPV, first prenatal visit; IADPSG, the International Association of the Diabetes and Pregnancy Study Group; PIH, pregnancy induced hypertension; BW >90^th^ percentile, a birth weight above the gender-specific 90^th^ percentile for gestational age; NICU, neonatal intensive care unit.

22. The conclusion is way too conclusive. The most you can say is that these data suggest an alternative method for early screening for GDM but needs to be prospectively validated in multiple ethnic cohorts and with appropriate reporting of perinatal outcomes. The authors should consider publishing only after external validation of their findings

*Ans*: Thanks for your suggestion. We have revised the manuscript according to your suggestions as below.

In Discussion (page 26-27, line 343-349): In conclusion, FPG at the FPV and the prevalence of GDM increase by age. By using maternal age plus FPG at the FPV, we suggest a way to diagnose GDM with reduced number of people taking OGTT. This may be especially useful in populations with a significant proportion of women getting pregnant until older ages. Our results provide an alternative screening strategy to previously published algorithms using the combination of various risk factors of GDM, such as history of GDM, family history of diabetes, ethnicity, parity, and BMI (30, 31).

Manuscript No.: PONE-D-20-00081

Title: Simplifying the gestational diabetes screen by maternal age plus fasting plasma glucose at first prenatal visit: a prospective cohort study

PLOS ONE

Responses to Reviewer #3:

Thank you very much for your constructive suggestions for this manuscript. We have made the revisions according to the comments, point by point, as follows. The page and line are according to revised manuscript with track changes.

**Abstract**

1. The abstract needs to state whether all women underwent fasting glucose screening (universal or risk factor based).

*Ans*: Thanks for your suggestion. We have revised the abstract according to your suggestions as below.

In Abstract (page 3, line 41-42): All participants underwent FPG and haemoglobin A1c (HbA1c) at the FPV and received 75-g OGTT at 24-28 gestational weeks if FPG at the FPV was <92 mg/dL.

2. The abstract also needs to describe what the OGTT eligibility criteria were (not including maternal age and fasting glucose)- this could be a brief statement just mentioning risk factor based or universal.

*Ans*: Thanks for your suggestion. We have revised the abstract according to your suggestions as below.

In Abstract (page 3, line 41-43): All participants underwent FPG and haemoglobin A1c (HbA1c) at the FPV and received 75-g OGTT at 24-28 gestational weeks if FPG at the FPV was <92 mg/dL. GDM was diagnosed by the IADPSG criteria.

3. It appears that 62% of pregnant women were OGTT screened at baseline: was this because certain thresholds of BMI or family history were used to determine eligibility for OGTT? Or did a large group of women decline screening by OGTT? The abstract needs to state which threshold was used for fasting glucose, and how this was determined, and which age threshold was applied- generally adding a little more numerical data on the paper’s findings.

*Ans*: In our study, all pregnant women with FPG at FPV <92 mg/dl received OGTT. The “FPG at the FPV” algorithm reduced OGTT% to 68.8%. (This number was 62.8% in the manuscript before revision. However, since another reviewer suggest exclusion of women with a history of GDM, the number became 68.8% in the revised manuscript.)

We have described the threshold of FPG, age plus FPG and how this was determined in abstract according to your suggestion.

In Methods of Abstract (page 3, line 41-45): All participants underwent FPG and haemoglobin A1c (HbA1c) at the FPV and received 75-g OGTT at 24-28 gestational weeks if FPG at the FPV was <92 mg/dL. GDM was diagnosed by the IADPSG criteria. Two algorithms were developed with the cutoffs determined when the percentage requiring OGTT (OGTT%) was the lowest and the sensitivity was ≥90%.

In Results of Abstract (page 3-4, line 46-49): The prevalence of GDM increased with age. The “FPG at the FPV” algorithm reduced OGTT% to 68.8% with the FPG cutoff at 79 mg/dl. The "age plus FPG at the FPV" algorithm, with the cutoff of 114, further reduced OGTT% to 58.3%, with the sensitivity of 90.7% (9.3% GDM missed) and the specificity of 100%.

4. Also, please add the number of diagnoses of GDM missed when the number of OGTTs is reduced by the use of maternal age, and fasting glucose.

*Ans*: Thanks for your suggestion. We have revised the manuscript according to your suggestions as below.

In Results of Abstract (page 3-4, line 46-49): The prevalence of GDM increased with age. The “FPG at the FPV” algorithm reduced OGTT% to 68.8% with the FPG cutoff at 79 mg/dl. The "age plus FPG at the FPV" algorithm, with the cutoff of 114, further reduced OGTT% to 58.3%, with the sensitivity of 90.7% (9.3% GDM missed) and the specificity of 100%.

**Introduction**

5. Needs to specify what the current recommendations are of guidelines (e.g. ADA, WHO) regarding universal or risk-factor based screening or prognostic model guided screening. To my knowledge, the guidelines to not specify a preference-this would be good to point out in the introduction. Using prognostic models is not a new approach.

*Ans*: We have revised the manuscript according to your suggestions.

In Introduction (page 5, line 59-71): For the diagnosis of gestational diabetes mellitus (GDM), the International Association of Diabetes and Pregnancy Study groups (IADPSG) and the World Health Organization (WHO) recommends the use of 75-g oral glucose tolerance test (OGTT) for all pregnant women at 24–28 weeks of gestation, which is also a diagnostic option suggested by the American Diabetes Association (ADA) in addition to the two-step method (1-3). Since the OGTT is burdensome for the pregnant women and the healthcare system, some screening methods have been developed, such as risk factor-based models or the 1-hour 50g glucose challenge test used in the two-step method (4). In addition, some studies have reported fasting plasma glucose (FPG)-based screening methods aimed at reducing the use of OGTTs (5-7). However, these FPG-based screening methods have generally been targeted at 24–28 weeks of gestation instead of early pregnancy, and the use of an FPG-based method at 24-28 weeks essentially requires two steps to diagnose or exclude GDM (5-8).

**Methods**

6. Could benefit from specifying which variables were eligible for the prognostic model; did BMI or family history or obstetric history feature in the multivariable logistic model?

*Ans*: We have revised the manuscript according to your suggestion. Age, FPG at FPV, HbA1c, family history of DM, and pre-pregnancy BMI were included in the multivariable logistic model.

In Methods (page 8, line 123-127): Logistic regression models were used to estimate the odds ratios of GDM for various risk factors. Variables associated with GDM in univariate logistic regression analyses were included in the multiple logistic regression analysis, including age, FPG at FPV, HbA1c, family history of DM, and pre-pregnancy BMI.

7. Also, needs to state that pregnant women who qualified for GDM diagnosis after just a fasting glucose, were not eligible to continue in the study.

*Ans*: Thanks for your suggestion. We have revised the manuscript to describe the diagnostic criteria of GDM more clearly.

In Methods (page 7-8, line 107-112): Eligible participants received blood tests for FPG and haemoglobin A1c (HbA1c) at the FPV after fasting for 8-10 hours. Women with FPG at the FPV less than 92 mg/dL received 75-g OGTT at 24-28 gestational weeks. The diagnosis of GDM was based on the recommendations of the IADPSG if FPG at the FPV ≥92 mg/dL, or if one of the results from the OGTT exceeded the cutoffs, including FPG ≥92 mg/dL, plasma glucose at 1h ≥180 mg/dL, or plasma glucose at 2h ≥153 mg/dL (1).

8. Was PCOS diagnosis available in this study, or was history of macrosomia available? These are additional risk factors that are sometimes used to specify eligibility for OGTT.

*Ans*: We have added PCOS and history of macrosomia added in Table 1. Besides, the unadjusted odds ratios of GDM were 1.1 (95% CI 0.31-3.86, p=0.89) for PCOS and 6 (95% CI 0.83-43, p=0.08) for history of macrosomia. Therefore, these two risk factors were not included in the multiple logistic regression model.

In Result (page 12, line 184-191):

Table 1. Clinical characteristics and laboratory test results at the first prenatal visit and OGTT result at 24-28 gestational weeks in pregnancy women with and without gestational diabetes mellitus (GDM) in the training cohort and validation cohort.

|  | The training cohort | | | The validation cohort | | |
| --- | --- | --- | --- | --- | --- | --- |
| Baseline characteristics | Non-GDM | GDM | P value | Non-GDM | GDM | P value |
| N | 437 (85.4 %) | 75 (14.6 %) | NA | 125 (80.2 %) | 26 (17.2 %) | NA |
| Age (years) | 33.5 (4.1) | 35.1 (4.1) | **0.002** | 33.3 (3.6) | 33.8 (2.9) | 0.55 |
| Age≥35 (N, %) | 190 (43.5 %) | 42 (56 %) | **0.04** | 46 (36.8 %) | 11 (42.3 %) | 0.32 |
| Nulliparous (N, %) | 142 (32.5 %) | 26 (34.7 %) | 0.68 | 46 (36.8 %) | 13 (50 %) | 0.48 |
| Gestational age at the FPV (weeks) | 10.1 (1.9) | 10.3 (2.2) | 0.81 | 10 (1.5) | 9.9 (1.5) | 0.58 |
| Family history of DM (N, %) | 96 (22 %) | 26 (34.7 %) | **0.02** | 21 (16.8 %) | 5 (19.2 %) | 0.81 |
| History of PCOS | 16 (3.7 %) | 3 (4 %) | 0.89 | 7 (5.6 %) | 3 (11.5 %) | 0.27 |
| History of macrosomia | 2 (0.5 %) | 2 (2.7 %) | 0.05 | 0 | 0 | - |
| Pre-pregnancy BW (kg) | 55.3 (8.4) | 57.8 (14) | **0.03** | 55.2 (8.1) | 60.2 (8.5) | **0.005** |
| Pre-pregnancy BMI (kg/m2) | 21.9 (3.3) | 22.9 (5.3) | **0.02** | 21.9 (3.2) | 23.7 (3.5) | **0.02** |
| **GWG at 24-28 gestational weeks (kg)** | **6.3 (3.7)** | **6.3 (2.7)** | **0.4** | **6.1 (3.2)** | **6.2 (2.3)** | 0.92 |
| Laboratory test results at the first prenatal visit | | | |  |  |  |
| FPG (mg/dL) | 81.1 (5) | 88 (7.4) | **<0.001** | 82.8 (4.7) | 90.9 (7.2) | **<0.001** |
| HbA1c (%) | 5.2 (0.2) | 5.4 (0.3) | **<0.001** | 5.1 (0.3) | 5.3 (0.3) | **<0.001** |
| Glucose level during OGTT at 24-28 gestational weeks | | | |  |  |  |
| FPG during OGTT (mg/dL) | 77.9 (5) | 82.3 (6.9) | **<0.001** | 78.8 (5) | 85.2 (5.4) | **<0.001** |
| 1hPG (mg/dL) | 128.2 (24.3) | 164.7 (29.5) | **<0.001** | 127.6 (27.3) | 149 (34.3) | **<0.001** |
| 2hPG (mg/dL) | 109.9 (19.9) | 150.2 (29.4) | **<0.001** | 108.6 (21.3) | 135.6 (39.7) | **<0.001** |

Mean (standard deviations) or N (%) were shown.

BMI, body mass index; BW, body weight; DM, diabetes mellitus; FPG, fasting plasma glucose; FPV, first prenatal visit; GDM, gestational diabetes mellitus; GWG, gestational weight gain; HbA1c, hemoglobin A1c; OGTT, oral glucose tolerance tests; FPG during OGTT, fasting plasma glucose during oral glucose tolerance tests; PCOS, Polycystic ovary syndrome; 1hPG, 1-hour plasma glucose during oral glucose tolerance tests; 2hPG, 2-hour plasma glucose during oral glucose tolerance tests

9. The comment ‘the specificity for all models was 100%, regardless of the threshold used’ is likely due to the way the model was established. Specificity is the probability that, given that someone has no GDM, the test would indicate that she had no GDM: given that the authors were looking at the effect of reducing the number of OGTTs performed, they are not going to find false positives (so lower specificity). This ‘self-fulfilling prophecy’ could be more clearly explained in the last paragraph of the methods’.

*Ans*: Thanks for your suggestion. We have revised the manuscript more clearly.

In Method (page 9, line 130-141): To search for the optimal cutoffs to identify those who do not need further OGTT in the second trimester, we calculated the performance of each algorithm. The percentage of women requiring OGTT (OGTT%) was calculated as follows: (the number of women whose FPG at FPV<92 mg/dl and FPG at the FPV or age plus FPG at the FPV ≥ the cutoff value) / (the number of whole population). In both algorithms, pregnant women were diagnosed as GDM either by FPG at the FPV≥92 mg/dL or the results of OGTT. Therefore, the false positive rate (FPR) was 0% for both algorithms. By definition, specificity equals to 1 – FPR, which means the specificity in all algorithms should ideally be 100%, no matter which cutoffs were chosen. Therefore, the determination of the optimal cutoffs was a trade-off between sensitivity and OGTT%. The optimal cutoff was chosen when the sensitivity was greater than 90% and the OGTT% was the lowest.

10. It is unclear why the authors have focused on only age and fasting glucose in their attempt to reduce the number of OGTTs, when more data is available. This needs to be elaborated.

*Ans*: We have revised the manuscript to describe why only age and fasting glucose were selected in Methods.

In Methods (page 8-9, line 123-130): Logistic regression models were used to estimate the odds ratios of GDM for various risk factors. Variables associated with GDM in univariate logistic regression analyses were included in the multiple logistic regression analysis, including age, FPG at FPV, HbA1c, family history of DM, and pre-pregnancy BMI. Then, two algorithms were constructed by the variables independently associated with GDM in the multivariate logistic regression model, including age and FPG at FPV. One algorithm made use FPG at the first prenatal visits alone, and the other included both age and FPG at the first prenatal visits.

**Results**

11. Given that almost half of women were excluded, it would be good to present a table with baseline characteristics of those with and without OGTT data available (so not twins or those with suspected pre-conception DM2), to establish generalizability of this study’s findings.

*Ans*: Thanks for your suggestion. We have added the results of the analysis in Results and S1 Table. There was no significant difference in clinical characteristics between these two groups.

In Result (page 11, line 169-172): In S1 Table, clinical characteristics between the 512 women with FPG and HbA1c at FPV and the 419 women without FPG and HbA1c at FPV were compared. There was no significant difference in clinical characteristics between these two groups.

S1 table. Clinical characteristics and laboratory test results at the first prenatal visit and OGTT result at 24-28 gestational weeks in pregnancy women with and without FPG and HbA1c at the first prenatal visit.

| Baseline characteristics | **With** FPG and HbA1c at the FPV | **Without** FPG and HbA1c at the FPV | P value |
| --- | --- | --- | --- |
| N | 512 | 407 |  |
| Age (years) | 33.8 (4.2) | 34 (4) | 0.36 |
| Age≥35 (N, %) | 232 (45.3 %) | 180 (44.2 %) | 0.63 |
| Nulliparous (N, %) | 168 (32.8 %) | 163 (40 %) | 0.68 |
| Gestational age at first recruitment (weeks) | 10.1 (2) | 26.5 (1.6) | - |
| Family history of DM (N, %) | 122 (23.8 %) | 79 (19.4 %) | 0.11 |
| History of PCOS | 19 (3.7 %) | 14 (3.4 %) | 0.83 |
| History of macrosomia | 4 (0.8 %) | 2 (0.5 %) | 0.59 |
| Pre-pregnancy BW (kg) | 55.6 (9.5) | 56.2 (9.2) | 0.34 |
| Pre-pregnancy BMI (kg/m2) | 22 (3.7) | 22 (3.3) | 0.89 |
| GWG at 24-28 gestational weeks (kg) | 6.3 (3.5) | 6.7 (3.3) | 0.07 |
| Glucose level during OGTT at 24-28 gestational weeks | | | |
| FPG during OGTT (mg/dL) | 79.2 (7) | 78.6 (5.5) | 0.13 |
| 1hPG (mg/dL) | 133.9 (29) | 133.6 (28.2) | 0.86 |
| 2hPG (mg/dL) | 115.5 (24.9) | 115.8 (25.8) | 0.86 |

Mean (standard deviations) or N (%) were shown.

BMI, body mass index; BW, body weight; DM, diabetes mellitus; FPG, fasting plasma glucose; FPV, first prenatal visit; GDM, gestational diabetes mellitus; GWG, gestational weight gain; HbA1c, aemoglobin A1c; OGTT, oral glucose tolerance tests; FPG during OGTT, fasting plasma glucose during oral glucose tolerance tests; PCOS, Polycystic ovary syndrome; 1hPG, 1-hour plasma glucose during oral glucose tolerance tests; 2hPG, 2-hour plasma glucose during oral glucose tolerance tests

12. Figure 1 states it reports the prevalence, when I think the authors mean incidence. Please amend.

*Ans*: We have revised the manuscript according to your suggestions. This figure is renumbered to be Figure 2.

In Result (page 14, line 198-199): Fig 2. The incidence of gestational diabetes mellitus (GDM) by age group. *** p for trend across all ages <0.001**

13. S2 Figure states that the ‘p for trend’ is starred, but does not specify for which comparison the star is presented: is this the trend across all ages, or the difference between highest and lowest age category.

*Ans*: Thanks for your suggestion. We have revised the legend according to your suggestions. The figure was re-numbered to S1 Figure in the revised version.

S1 Fig. Fasting plasma glucose (FPG) at the first prenatal visit by age group. *** p for trend across all age groups <0.05.**

14. Figure 2 presents the number of OGTTs in populations of varying age distributions. The models used to create this figure are not described in the methods, and must be added. Presumably, there would be some uncertainty (SD or 95% confidence interval) which could be better demonstrated in the figure.

*Ans*: The percentage of women requiring OGTT (OGTT%) was calculated as follows: (the number of women whose FPG at FPV<92 mg/dl and FPG at FPV or age plus FPG at the FPV ≥ the cutoff value) / (the number of whole population). Therefore, there was no uncertainty for OGTT% with a specific cutoff value. We have revised the manuscript to make it clearer.

In Method (page 9, line 132-135): The percentage of women requiring OGTT (OGTT%) was calculated as follows: (the number of women whose FPG at the FPV<92 mg/dl and FPG at the FPV or age plus FPG at the FPV ≥ the cutoff value) / (the number of whole population).

15. In line 158, at the end of the results, the actual age threshold used need to be added to the text. Did the authors toggle the age threshold, as they did for fasting glucose?

*Ans*: We have added some description for the actual age threshold in the “age plus FPG at the FPV” algorithm in Results. Besides, we did not toggle age threshold in the study.

In Results (page 14-15, line 209-215): The optimal cutoff value for FPG at the FPV in the "FPG at the FPV" algorithm was 79 mg/dl. Besides, the optimal cutoff value for age plus FPG at the FPV in the “age plus FPG at the FPV” algorithm was 114. By the cutoff of 114, if a 30-year-old woman whose FPG at the FPV was greater than 84 mg/dl, she should receive OGTT by the "age plus FPG at the FPV" algorithm, since her age plus FPG at FPV exceeded 114. Similarly, if a 35-year-old woman whose FPG at the FPV was greater than 79 mg/dl, then she should receive OGTT.

16. The last sentence of the results (line 159-160) now reads: the addition of FPG could reduce OGTT from 62 to 52%, this is strictly speaking not correct. The reduction from the current policy describe in the methods, is 100% OGTT to 52% OGTT screening when maternal age>=35 years and FPG> =80 are applied as eligibility criteria.

*Ans*: Thanks for your suggestion. We have deleted the sentence and revised the manuscript to make it clearer.

Besides, since another reviewer suggest the exclusion of women with a history of GDM, the analyses were re-performed. The findings were similar with a slight change in the numbers.

In Result (page 16, line 231-236): In the training cohort, we found that the percentage of women who required OGTT was reduced to 68.8% by the “FPG at the FPV” algorithm. Compared to the “FPG at the FPV” algorithm, the “age plus FPG at the FPV” algorithm further reduced OGTT% to 58.5%. Similarly, the OGTT% was decreased to 73.6% by the “FPG at the FPV” algorithm and 61.5% by the “age plus FPG at the FPV” algorithm in the validation cohort (Table 3).

17. The methods need to show whether women with a history of GDM were also excluded from OGTT screening if they were neither past 35 or had fasting glucose <80. I am assuming this is the case, but due to the rather large impact on chance of GDM, this needs to be explicitly stated.

*Ans*: We agree with you that a history of GDM is an important risk factor of GDM, and women with a history of GDM should receive OGTT to confirm the diagnosis. In addition, another reviewer suggest the exclusion of women with a history of GDM. Therefore, we have excluded these women, re-performed the analyses, and revised the manuscript.

In Methods (page 7, line 104-107): Since it is reasonable for pregnant women with a history of GDM to undergo OGTT, we excluded women with a history of GDM from the analyses in this study (women with history of GDM, N=35 in the training cohort, and N=7 in the validation cohort).

**Minor comments**

18. Generally, the English is fine, although it could be improved by English language editing. Examples include (Introduction) ‘…higher first-trimester FPG levels in early pregnancy is actually poorly….’ Which should be ‘…are actually poorly…’;

*Ans*: We have revised the manuscript according to your suggestions. Besides, we have sent the manuscript for English language editing. The proof is attached.

In Introduction (page 6, line 76-79): Since a higher first-trimester FPG level are associated with an increased risk of GDM diagnosed at 24-28 weeks (10), the FPG at the FPV may be a good predictor to be used in the screening of GDM.

19. (line 92 methods) ‘….All pregnant women were done with FPG…’ Which should be ‘…all pregnant women underwent FPG …’

*Ans*: We have revised the sentence.

In Method (page 7, line 107-108): Eligible participants received blood tests for FPG and haemoglobin A1c (HbA1c) at the FPV after fasting for 8-10 hours.

20. (line 102 methods) ‘… Maternal blood samples without fasting were excluded…’ which should be ‘… we excluded maternal blood samples of participants who had not fasted before the blood sample was drawn…’

*Ans*: Thanks for your suggestion. We found that this sentence was not meaningful, since all the women in the study received blood tests for FPG at FPV and FPG during OGTT had fasted for 8-10 hours. Therefore, we have deleted the sentence and rephrase another sentence describing the duration of fasting.

In Methods (page 7, line 107-108): Eligible participants received blood tests for FPG and haemoglobin A1c (HbA1c) at the FPV after fasting for 8-10 hours.

21. Supporting files flow chart ‘preformed’ which should be ‘performed’

*Ans*: We have corrected the typo.
