# Supplementary material for: Simplifying the screening of gestational diabetes by maternal age plus fasting plasma glucose at first prenatal visit: A prospective cohort study
Source: PLoS One. 2020 Aug 20;15(8):e0237224. doi: 10.1371/journal.pone.0237224 (PMC7444589; doi:10.1371/journal.pone.0237224)
Supplement: Attachment — Submitted filename: Response to Reviewers.docx [file pone.0237224.s007.docx]

Dear Editor-in-chief,

Thank you very much for offering us a chance to revise our manuscript and giving us the constructive comments. We have revised according to reviewers’ suggestions, point by point. All the questions raised by the reviewers are carefully addressed, please see below, and all changes are marked in the revised manuscript.

Thank you for your help in processing our manuscript.

Yours sincerely,

Shin-Yu Lin, M.D., Ph.D.

Assistant Professor, Department of Internal Medicine,

National Taiwan University Hospital

7 Chung-Shan South Road, Taipei, Taiwan.

Phone: +886-2-23123456 ext. 70919

E-mail: lin.shinyu@ntu.edu.tw

Manuscript No.: PONE-D-20-00081R1

Title: Simplifying the gestational diabetes screen by maternal age plus fasting plasma glucose at first prenatal visit: a prospective cohort study

PLOS ONE

Responses to Reviewer #2:

Thank you very much for your constructive suggestions for this manuscript. We have made the revisions according to the comments, point by point, as follows. The page and line are according to revised manuscript with track changes.

1. Introduction line 75 : should be …FPG level is associated

*Ans*: We have revised the manuscript according to your suggestion.

In Introduction (page 5-6, line 74-76): Since a higher first-trimester FPG level **is associated** with an increased risk of GDM diagnosed at 24-28 weeks (10), the FPG at the FPV may be a good predictor to be used in the screening of GDM.

2. From the new manuscript I now see that only those that had FPG at FPV of < 92 had a 24-28 week 75 gram OGTT. I thus assume that those with FPG > 91 were treated as GDM. Please clarify. If these women were in fact treated as GDM, this makes it very difficult to calculate the performance of age+FPG at FPV in diagnosing later GDM. In order to do this, FPG at FPV would be drawn in a blinded fashion (unless overt diabetes was found) and all would have an unblinded 75 gram OGTT at 24-28 weeks. Thus any conclusions from this study are only applicable to women with FPG < 92 at booking. This needs to be stated in the limitations as many countries do not view a FPG of >91 at FPV as synonymous with a diagnosis of GDM.

*Ans*: Thanks for your suggestion. In this study, pregnant women in the first trimester with fasting blood glucose value ≥ 92 mg/dl and <126 mg/dl were diagnosed as gestational diabetes according to the IADPSG guidelines. They were treated and did not receive OGTT in the second trimester. Only the women with the 1^st^ trimester fasting plasma glucose < 92 mg/dl received OGTT. Therefore, our conclusions were only applicable when GDM was diagnosed by the IADPSG criteria. We have added this in the limitation.

In the limitation (page 24, line 335-340): Secondly, pregnant women with FPG at FPV ≥ 92 mg/dl and <126 mg/dl were diagnosed as GDM according to the IADPSG guidelines, in addition to the criteria by the results of OGTT at 24-28^th^ gestational weeks. Since the diagnostic criteria for GDM varied in different countries, we have to emphasize that the conclusions of the present study are only applicable when GDM is diagnosed by the IADPSG criteria.

3. Methods Line 108: What does it mean the clinicians were “blinded to the results of the statistical analysis” The analysis was done retrospectively after the cohorts were formed so this would not be relevant. What is relevant, is to indicate that clinicians were not blinded to the FPG at FPV

*Ans*: We have deleted the sentence and revised the manuscript according to your suggestions.

In Methods (page 7, line 108-109): The clinicians were not blinded to the results of FPG at FPV and OGTT.

4. Methods line 125 “One algorithm made use FPG…” revise the sentence

*Ans*: We have revised the manuscript according to your suggestion.

In Introduction (page 8, line 124-126): One algorithm **used** FPG at the first prenatal visits alone, and the other included both age and FPG at the first prenatal visits.

5. Methods lines 131 – 134, Discussion 304-306, Table 2 and Figure 3. I need to disagree with the statement that specificity was 100%. If one is doing a FPG and FPV (diagnosing GDM at FPV on all above arbitrary threshold) and then a GTT (diagnostic test) in all the remaining, then one is NOT doing screening but rather universal diagnostic testing with, by design, 100% sensitivity and 100% specificity in the entire cohort. Thus, do to the study design, the specificity was 100% in the cohort but this would not be true for the models using different thresholds for FPG at FPV. In your models I believe you are actually attempting to answer the question: how does FPV FPG below the diagnostic threshold perform as a screening test for 24-28 week GDM compared with FPG+Age at FPV? Based on Figure 3 the specificity is definitely not 100%. In both algorithms 59.9% and 49.4% that go on to have the OGTT based on the FPG or FPG+age cutoffs respectively, are found NOT to have GDM and thus are FALSE POSITIVES for the FPG screening test and the outcome of GDM. From the figure – there is no information on the false negatives i.e those below the threshold (FPG or FPG+Age) that were diagnosed with GDM. We know that number is not 0 as in the results (Table 2) the sensitivity is < 100%. One can design the algorithms to perform at a prespecified sensitivity or specificity (even a theoretical 100%) but always (unless you have found the perfect screening test when compared to the gold standard), high specificity will be at the expense of sensitivity and vice versa. Neither the false negatives or the false positives are correctly indicated in the figure or in the text and thus the interpretation of this study appears problematic. I think that in the algorithms the authors need to reflect on their assumptions regarding specificity and sensitivity or at least recheck the numbers. Once this is sorted out – reporting a ROC curve and the PPV and NPV would be useful.

*Ans*: We have added a paragraph in Results and S2 Figure to better illustrate how the performance of the algorithms were calculated.

Table 2 summarized the performance of different cutoffs in the two algorithms.

In Result (page 16-17, line 234-249): To better illustrate how the performance of the algorithms were calculated, we have added the numbers of women (N) in both algorithms (S2 Fig). In the "FPG at the FPV" algorithms, GDM was diagnosed by FPG at the FPV ≥ 92 mg/dl (N=29) or the results of OGTT when FPG at FPV ≥79 mg/dl (N=40). FPG at the FPG ≥92 mg/dl and the results of OGTT are both diagnostic criteria of GDM by the IADPSG. No matter what the cutoff was chosen, the results of OGTT could correctly diagnose GDM. Therefore, there was no false positive by the algorithm, and the specificity of the algorithm was 100%. On the other hand, the "FPG at the FPV" algorithm excluded GDM if FPG <79 mg/dl (N=131) or by the results of OGTT when FPG at FPV ≥79 mg/dl (N=312). There were 6 women with GDM by the IADPSG criteria who were classified as "GDM excluded" (false negatives). Therefore, the sensitivity was 69/(6+69), which was equal to 92%. Since the results of OGTT could always correctly diagnose GDM by the IADPSG criteria, the false negatives came from women with FPG <79 mg/dl. If the FPG at the FPV cutoff was higher, there would be more false negatives (lower sensitivity) and less OGTT performed. The performance of the "age plus FPG at the FPV" algorithm was calculated similarly.

S2 Fig. Algorithms to screen gestational diabetes mellitus (GDM) by (A) fasting plasma glucose (FPG) at the first prenatal visit (FPV) and (B) age plus FPG at the FPV. The number of pregnant women in different paths are shown.

Table 2. Performance for different cutoffs to screen gestational diabetes mellitus (GDM) by the “FPG at the FPV” algorithm and "age plus FPG at the FPV" algorithm.

| FPG at the FPV | | | |  | Age plus FPG at the FPV | | | |
| --- | --- | --- | --- | --- | --- | --- | --- | --- |
| Cutoffs to exclude GDM | OGTT (%) | Sensitivity (%) | Specificity (%) |  | Cutoffs to exclude GDM | OGTT (%) | Sensitivity (%) | Specificity (%) |
| 77 | 78.1 % | 96 % | 100% |  | 112 | 66.2 % | 93.3 % | 100% |
| 78 | 72.9 % | 96 % | 100% |  | 113 | 62.1 % | 90.7 % | 100% |
| **79** | **68.8 %** | **92 %** | **100%** |  | **114** | **58.3 %** | **90.7 %** | **100%** |
| 80 | 63.8 % | 89.3 % | 100% |  | 115 | 53.5 % | 89.3 % | 100% |

6. Methods – line 140-143: language is unclear

*Ans*: We have revised the language to make it clearer.

In Methods (page 9-10, line 138-148): For every cutoff to exclude GDM, the prevalence of GDM, OGTT% and sensitivity values in women younger than 35 years (prevalence_<35y/o_, OGTT%_<35y/o_, and sensitivity_<35y/o_) and in women older than 35 years (prevalence_≥35y/o_, OGTT%_≥35y/o_, and sensitivity_≥35y/o_) were calculated separately. The OGTT% and sensitivity in the whole population were calculated assuming the percentage of women older than 35 years (percentage_≥35y/o_) were 0%, 10%, 20%, etc., to 100%. For each percentage_≥35y/o_, OGTT% in the whole population was calculated as follows: (percentage_≥35y/o_ * OGTT%_≥35y/o_ + (1 - percentage_≥35y/o_) * OGTT%_<35y/o_). For each percentage_≥35y/o_, sensitivity in the whole population was calculated as follows: (percentage_≥35y/o_ * prevalence_≥35y/o_ * sensitivity_≥35y/o_ + (1 - percentage_≥35y/o_) * prevalence_<35y/o_ * sensitivity_<35y/o_) / (percentage_≥35y/o_ * prevalence_≥35y/o_ + (1 - percentage_≥35y/o_) * prevalence_<35y/o_).

7. Results Line 151-155 – you are repeating methodology – should not be in the results

*Ans*: Thanks for your suggestion. We have deleted the sentences according to your suggestion.

8. Table 1: Ideally there would be a comparison of the characteristics of the two cohorts to demonstrate that over time there was no change in population characteristics

*Ans*: We have revised the manuscript according to your suggestion.

In Result (page 11, line 177-182): Besides, we have compared clinical characteristics in women with or without GDM between the training cohort and validation cohort. Women without GDM in the validation cohort had a slightly higher FPG and slightly lower HbA1c than women without GDM in the training cohort. In the women with GDM, 1-hour plasma glucose during OGTT at 24-28 gestational weeks were lower in the validation cohort than that in the training cohort.

In Result (page 12-13, line 184-192):

Table 1. Clinical characteristics and laboratory test results at the first prenatal visit and OGTT result at 24-28 gestational weeks in pregnancy women with and without gestational diabetes mellitus (GDM) in the training cohort and validation cohort.

|  | The training cohort | | | The validation cohort | | |
| --- | --- | --- | --- | --- | --- | --- |
| Baseline characteristics | Non-GDM | GDM | P value | Non-GDM | GDM | P value |
| N | 437 (85.4 %) | 75 (14.6 %) |  | 125 (80.2 %) | 26 (17.2 %) |  |
| Age (years) | 33.5 (4.1) | 35.1 (4.1) | **0.002** | 33.3 (3.6) | 33.8 (2.9) | 0.55 |
| Age≥35 (N, %) | 190 (43.5 %) | 42 (56 %) | **0.04** | 46 (36.8 %) | 11 (42.3 %) | 0.32 |
| Nulliparous (N, %) | 142 (32.5 %) | 26 (34.7 %) | 0.68 | 46 (36.8 %) | 13 (50 %) | 0.48 |
| Gestational age at the FPV (weeks) | 10.1 (1.9) | 10.3 (2.2) | 0.81 | 10 (1.5) | 9.9 (1.5) | 0.58 |
| Family history of DM (N, %) | 96 (22 %) | 26 (34.7 %) | **0.02** | 21 (16.8 %) | 5 (19.2 %) | 0.81 |
| History of PCOS | 16 (3.7 %) | 3 (4 %) | 0.89 | 7 (5.6 %) | 3 (11.5 %) | 0.27 |
| History of macrosomia | 2 (0.5 %) | 2 (2.7 %) | 0.05 | 0 | 0 | - |
| Pre-pregnancy BW (kg) | 55.3 (8.4) | 57.8 (14) | **0.03** | 55.2 (8.1) | 60.2 (8.5) | **0.005** |
| Pre-pregnancy BMI (kg/m2) | 21.9 (3.3) | 22.9 (5.3) | **0.02** | 21.9 (3.2) | 23.7 (3.5) | **0.02** |
| GWG at 24-28 gestational weeks (kg) | 6.3 (3.7) | 6.3 (2.7) | 0.4 | 6.1 (3.2) | 6.2 (2.3) | 0.92 |
| Laboratory test results at the first prenatal visit | | | |  |  |  |
| FPG (mg/dL) | 81.1 (5) | 88 (7.4) | **<0.001** | 82.8 (4.7)* | 90.9 (7.2) | **<0.001** |
| HbA1c (%) | 5.2 (0.2) | 5.4 (0.3) | **<0.001** | 5.1 (0.3)* | 5.3 (0.3) | **<0.001** |
| Glucose level during OGTT at 24-28 gestational weeks | | | |  |  |  |
| FPG during OGTT (mg/dL) | 77.9 (5) | 82.3 (6.9) | **<0.001** | 78.8 (5) | 85.2 (5.4) | **<0.001** |
| 1hPG (mg/dL) | 128.2 (24.3) | 164.7 (29.5) | **<0.001** | 127.6 (27.3) | 149 (34.3)**†** | **<0.001** |
| 2hPG (mg/dL) | 109.9 (19.9) | 150.2 (29.4) | **<0.001** | 108.6 (21.3) | 135.6 (39.7) | **<0.001** |

Mean (standard deviations) or N (%) were shown. BMI, body mass index; BW, body weight; DM, diabetes mellitus; FPG, fasting plasma glucose; FPV, first prenatal visit; GDM, gestational diabetes mellitus; GWG, gestational weight gain; HbA1c, hemoglobin A1c; OGTT, oral glucose tolerance tests; FPG during OGTT, fasting plasma glucose during oral glucose tolerance tests; PCOS, Polycystic ovary syndrome; 1hPG, 1-hour plasma glucose during oral glucose tolerance tests; 2hPG, 2-hour plasma glucose during oral glucose tolerance tests.

*** p<0.05 vs. women without GDM in the training cohort.**

**† p<0.05 vs. women with GDM in the training cohort.**

9. Results will be easier to follow if arranged with subheadings

*Ans*: Thanks for your suggestion. We have added subheadings in the Result section, including "*The Training Cohort and The Validation Cohort*", "*The Incidence of GDM by Age*", "*The development of the screening algorithms for GDM*", and "*The impact of age*".

10. Table 4. The outcome analysis. The comparison should not be between outcomes in women with and without GDM (as obviously the rate will be higher in GDM) but rather between rate of outcomes in each cohort compared with gold standard (IADPSG). Due to the way this study was designed the results are not representative of true perinatal risk as NO GDMs were undiagnosed and untreated, which is what would happen if using a first trimester screening test with < 100% sensitivity. Based on this this table can likely be omitted and replaced with a statement that “ The impact of adopting either algorithm needs to be evaluated in future prospective blinded trials”

*Ans*: We agree with you, and we have deleted Table 4. Besides, we have also deleted S3 Table, since this table described the pregnancy outcomes in women with false negative results and may have similar problem.

11. Discussion: Line 328-330. Actually I do not believe that not having GWG at the FPV is significant. There is minimal weight gain in the first 10 weeks of pregnancy thus the impact would be minimal. Adjusting for GWG up until the 24-28 week GTT is relevant

*Ans*: We agree with you, and we have deleted the sentence.
