# Supplementary material for: Simplifying the screening of gestational diabetes by maternal age plus fasting plasma glucose at first prenatal visit: A prospective cohort study
Source: PLoS One. 2020 Aug 20;15(8):e0237224. doi: 10.1371/journal.pone.0237224 (PMC7444589; doi:10.1371/journal.pone.0237224)
Supplement: Attachment — Submitted filename: Simplifying the gestational diabetes screen BERGER revision 2.docx [file pone.0237224.s008.docx]

Simplifying the gestational diabetes screen 1 by maternal age plus fasting plasma glucose at first prenatal visit: a prospective cohort study

The authors present a cohort study aimed at devising a strategy to decrease the need for OGTT at 24-28 weeks gestation based on the combination of maternal age and fasting plasma glucose (FPG) at the first prenatal visit (FPV). This is a subject that has practical clinical applicability and could be useful in clinical settings if proven safe and effective. Many studies have attempted to create predictive models in early pregnancy to predict mid trimester GDM. Most are complex and/or have poor sensitivity, specificity or both. My comments regarding the current study can be found below. The paper will benefit from additional grammatical revision

1. I don’t understand the subtitle: “Early screen of gestational diabetes: role of maternal age” appears to be redundant or inserted by mistake.

Abstract:

2. As below – FPG at FPV is not universally performed or recommended
3. 547 were not consecutively recruited as > 1000 were eligible and approached

Introduction

4. line 54-57: Inaccurate - ADA has allowed the OPTION of one step 75 gram OGTT but also allow the wo step 50 g GCT/100 gram GTT
5. FPG at first visit is NOT universally recommended by ADA – only in those with risk factors. Even in those the recommendation is to test using “standard criteria”
6. Line 64 – 66 again, FPG not universally performed at FPV or first trimester (likely the same)
7. Line 67-69 – sentence contradicts itself if “poorly predictive” of GDM in early pregnancy why would FPG at the FPV (which is also early pregnancy) be a “good predictor” ?
8. Line 70: Really? Age is more independently important than obesity, family history previous GDM etc?

Methods

9. If I understand correctly, women were recruited at FPV and were eligible if they were singleton, viable and had no known preexisting DM. Then they had FPG and A1C. If they had PDM after these tests they were excluded. All that remained had a OGTT at 24-28 weeks. This should be clarified and shown in a flow diagram with numbers as Fig 1 (not as supplementary material)
10. From figure S1 – I note that nearly half of the base population did not have FPG and A1C. In line 92 you say that “ALL pregnant women were done with FPG and hemoglobin A1c (HbA1c)…” Please clarify this. Was in fact FPG and A1C a standardized protocol at the time of the study? If so why did > 400 women not have this done. Were they perhaps different clinically than those that did? Were all 1065 approached and consented if singleton and no PDM? If so – did the 419 without FPG and A1C not consent? This is all confusing and needs to be clarified.
11. Was there a gestational age criteria for the FPV? If someone had a FPV at 20 weeks was she included? Please clarify
12. When defining BMI was prepregnancy BMI used? Early pregnancy?
13. Were the FPG and A1C results available to the clinicians? What was done if at FPV the fPG was > 5.1 or A1C as 5.9-6.4?

Results

14. Was gestational weight gain collected and if not – why?
15. Why is FPG of >91 at FPV (10 weeks mean) diagnostic of GDM (Fig 2)?? This is not standard. The IADPSG values are extrapolated from HAPO which used GTT values at 24-28 weeks. There are no outcome based validated thresholds for early GTT. Were these women treated (see comment 11)
16. Line 142-143: In addition to age, previous GDM was also independently associated with GDM OR 8.14. This is not mentioned. Also what about the effect of BMI? It was significant on the univariate analysis and it is mentioned in the legend (and in line 232 of the discussion) but not in the body of the table. What happened to this variable?

Discussion

17. Discussion overall VERY repetitive.
18. Line 227 – provide references for evidence that early identification of “GDM” leads to better outcomes
19. These results are not consistent with the study by Zhu et al DIABETES CARE, VOLUME 36, MARCH 2013. In this study even if FPG at FPV as < 4.1 8.1% had a subsequent diagnosis of GDM at 24-28 weeks. In addition they were able to show that overall – there was poor correlation between the FPG at FPV and the FPG at the OGTT. 100% specificity was achieved only > 5.7 (large) study’s results and yours. In addition discuss why FPG performs favorably as a screening tool in this study in contrast with what is known regarding the poor performance of early FPG as a screening tool for future GDM (See summary of evidence in NICE guidelines).
20. Missing references for other predictive models including recent: Zheng, T., Ye, W., Wang, X. et al. A simple model to predict risk of gestational diabetes mellitus from 8 to 20 weeks of gestation in Chinese women. BMC Pregnancy Childbirth 19, 252 (2019). <https://doi.org/10.1186/s12884-019-2374-8>.
21. Missing many of the other limitations:
    a. The “missing” 419 women that did not have FPG and A1C at FPV. If you can show that their characteristics are not different from those that had testing then that will provide some support for the validity of the results
    b. The lack of clinician blinding
    c. Unlike the authors previous study – there is no validation cohort
    d. No reporting of gestational weight gain
    e. No reporting of perinatal outcomes. What are the outcomes for those missed by this strategy (90% sensitivity compared with 100% sensitivity with universal OGTT)
22. The conclusion is way too conclusive. The most you can say is that these data suggest an alternative method for early screening for GDM but needs to be prospectively validated in multiple ethnic cohorts and with appropriate reporting of perinatal outcomes.

Revision:

I would like to thank the authors for the thoughtful revisions. I still have significant reservations regarding the data presented with regards to specificity and sensitivity of the models (see comment 5) General: Some additional grammatic editorial work is needed, especially in the parts that were revised.

1. Introduction line 75 : should be …FPG level is associated
2. From the new manuscript I now see that only those that had FPG at FPV of < 92 had a 24-28 week 75 gram OGTT. I thus assume that those with FPG > 91 were treated as GDM. Please clarify. If these women were in fact treated as GDM, this makes it very difficult to calculate the performance of age+FPG at FPV in diagnosing later GDM. In order to do this, FPG at FPV would be drawn in a blinded fashion (unless overt diabetes was found) and all would have an unblinded 75 gram OGTT at 24-28 weeks. Thus any conclusions from this study are only applicable to women with FPG < 92 at booking. This needs to be stated in the limitations as many countries do not view a FPG of >91 at FPV as synonymous with a diagnosis of GDM.
3. Methods Line 108: What does it mean the clinicians were “blinded to the results of the statistical analysis” The analysis was done retrospectively after the cohorts were formed so this would not be relevant. What is relevant, is to indicate that clinicians were not blinded to the FPG at FPV
4. Methods line 125 “One algorithm made use FPG…” revise the sentence
5. Methods lines 131 – 134, Discussion 304-306, Table 2 and Figure 3. I need to disagree with the statement that specificity was 100%. If one is doing a FPG and FPV (diagnosing GDM at FPV on all above arbitrary threshold) and then a GTT (diagnostic test) in all the remaining, then one is NOT doing screening but rather universal diagnostic testing with, by design, 100% sensitivity and 100% specificity in the entire cohort. Thus, do to the study design, the specificity was 100% in the cohort but this would not be true for the models using different thresholds for FPG at FPV. In your models I believe you are actually attempting to answer the question: how does FPV FPG below the diagnostic threshold perform as a screening test for 24-28 week GDM compared with FPG+Age at FPV? Based on Figure 3 the specificity is definitely not 100%. In both algorithms 59.9% and 49.4% that go on to have the OGTT based on the FPG or FPG+age cutoffs respectively, are found NOT to have GDM and thus are FALSE POSITIVES for the FPG screening test and the outcome of GDM. From the figure – there is no information on the false negatives i.e those below the threshold (FPG or FPG+Age) that were diagnosed with GDM. We know that number is not 0 as in the results (Table 2) the sensitivity is < 100%. One can design the algorithms to perform at a prespecified sensitivity or specificity (even a theoretical 100%) but always (unless you have found the perfect screening test when compared to the gold standard), high specificity will be at the expense of sensitivity and vice versa. Neither the false negatives or the false positives are correctly indicated in the figure or in the text and thus the interpretation of this study appears problematic. I think that in the algorithms the authors need to reflect on their assumptions regarding specificity and sensitivity or at least recheck the numbers. Once this is sorted out – reporting also a ROC curve and the PPV and NPV would be useful.
6. Methods – line 140-143: language is unclear
7. Results Line 151-155 – you are repeating methodology – should not be in the results
8. Table 1: Ideally there would be a comparison of the characteristics of the two cohorts to demonstrate that over time there was no change in population characteristics
9. Results will be easier to follow if arranged with subheadings
10. Table 4. The outcome analysis. The comparison should not be between outcomes in women with and without GDM (as obviously the rate will be higher in GDM) but rather between rate of outcomes in each cohort compared with gold standard (IADPSG). Due to the way this study was designed the results are not representative of true perinatal risk as NO GDMs were undiagnosed and untreated, which is what would happen if using a first trimester screening test with < 100% sensitivity. Based on this this table can likely be omitted and replaced with a statement that “ The impact of adopting either algorithm needs to be evaluated in future prospective blinded trials”
11. Discussion: Line 328-330. Actually I do not believe that not having GWG at the FPV is significant. There is minimal weight gain in the first 10 weeks of pregnancy thus the impact would be minimal. Adjusting for GWG up until the 24-28 week GTT is relevant

Revision 2 June 20

1. After all the revisions, the paper needs additional English language editing (e.g “The pregnant women in the validation cohort *was* recruited..” “Their clinical characteristics were acquired by questionnaire and recorded such as age, parity...”)
2. Page 10 line 163 – repeats page 10 line 156-157. Repeat description of validation cohort
3. Page 16 line 238: TYPO “FPG at the FPG ≥92 mg/dl and the results of OGTT are both…”
4. Table 1: Title Suggest instead of ” Clinical characteristics and laboratory test results at the first prenatal visit and OGTT result at 24-28 gestational weeks in *pregnancy* women with and without gestational diabetes mellitus (GDM) in the training cohort and validation cohort” - should be simplified to “Clinical characteristics and laboratory test results in *pregnant* women with and without gestational diabetes mellitus (GDM) in the training and validation cohort*”* Also add n=XX under the column headings
5. From table 1 – you appropriately highlight the small differences in blood sugar results between the validation and derivation cohort. This should be mentioned as a possible limitation to the validation portion of the analysis.
6. The prevalence of GDM is quite high – overall 14.6%. Does this seem reasonable for a universally tested Taiwanese population? If not – please address.
7. The authors state in the discussion that “the algorithms used to screen GDM proposed in the study are simple, practical, and can be used clinically”. This is true but it needs to be mentioned that it requires that all women have a fasting glucose test early in pregnancy. This is often difficult logistically and unpleasant for women that are likely experiencing nausea and vomiting. This can be mentioned as a drawback to this strategy.
8. The discussion would usually start with a summary statement of the major findings. This is not the case here.
9. Finally, I need to come back to the statement that there are no false positives and thus the specificity is 100%. See 2X2 table for the screening strategy of FPG at FPV using a threshold of 79 in all women with FPG <92 at FPV and using the values from S2 Fig:

|  | GDM+ | GDM- |  |
| --- | --- | --- | --- |
| FPG <79 | 6 | 125 | 131 |
| FPG ≥79 | 40 | 312 | 352 |
|  | 46 | 437 | 483 |

Sensitivity: 40/46= 86.9%

Specificity: 125/437 = 28.6%

NPV=125/131 = 95.4%

PPV= 40/352 = 11.4%

FPR: 312/437 = 71.4%

If you include those with FPG at FPV ≥ 92 results are as follows:

|  | GDM+ | GDM- |  |
| --- | --- | --- | --- |
| FPG < 79 | 6 | 125 | 131 |
| FPG ≥ 79 | 69 | 312 | 381 |
|  | 75 | 437 | 512 |
|  |  |  |  |
| Sens | 0.92 |  |  |
| Spec | 0.29 |  |  |
| NPV | 0.95 |  |  |
| PPV | 0.18 |  |  |
| FPR | 0.71 |  |  |

Thus, the statement (throughout the paper) that specificity is 100% is misleading. When everyone does FPG at FPV and ALL go on to have an OGTT (unless diagnosed by the FPG at FPV) then of course sensitivity and specificity are 100% but this type of strategy constitutes universal testing. In the model you are suggesting, only those with FPG <92 and greater than 79 would go on to have an OGTT. Most of those that screen “positive” (FPG >79) and thus have an OGTT do not have GDM and thus the PPV is low. Conversely, the majority of those without GDM screen positive at the FPV (312/437) and thus the specificity is 28.6% (FPR 71.4%). The low specificity is to be expected and is not necessarily problematic as this strategy still allows significantly fewer women to undergo a GTT at 24-28 weeks. Unless I am completely misunderstanding your explanation – this issue of specificity needs to be corrected throughout the paper.
