# Supplementary material for: Simplifying the screening of gestational diabetes by maternal age plus fasting plasma glucose at first prenatal visit: A prospective cohort study
Source: PLoS One. 2020 Aug 20;15(8):e0237224. doi: 10.1371/journal.pone.0237224 (PMC7444589; doi:10.1371/journal.pone.0237224)
Supplement: Attachment — Submitted filename: Response to Reviewers.docx [file pone.0237224.s009.docx]

Manuscript No.: PONE-D-20-00081R3

Title: Simplifying the gestational diabetes screen by maternal age plus fasting plasma glucose at first prenatal visit: a prospective cohort study

PLOS ONE

Responses to Reviewer #2:

Thank you very much for your constructive suggestions for this manuscript. We have made the revisions according to the comments, point by point, as follows. The page and line are according to revised manuscript with track changes.

1. After all the revisions, the paper needs additional English language editing (e.g “The pregnant women in the validation cohort was recruited..” “Their clinical characteristics were acquired by questionnaire and recorded such as age, parity...”)

*Ans*: We have revised the manuscript according to your suggestion.

In Research Design and Methods (page 7, line 101-102): The pregnant women in the validation cohort **were** recruited in 2019.

In Research Design and Methods (page 7, line 115-118): Their clinical characteristics were acquired by questionnaire**s**, such as age, parity, pre-pregnancy BMI, history of GDM and polycystic ovary syndrome (PCOS), family history of diabetes (defined as first degree relative with diabetes mellitus), etc.

2. Page 10 line 163 – repeats page 10 line 156-157. Repeat description of validation cohort

*Ans*: We have deleted the sentence and revised the manuscript according to your suggestions.

3. Page 16 line 238: TYPO “FPG at the FPG ≥92 mg/dl and the results of OGTT are both…”

*Ans*: We have revised the manuscript according to your suggestion.

In Result (page 16, line 246): FPG at the FPV ≥92 mg/dl and the results of OGTT are both diagnostic criteria of GDM by the IADPSG.

4. Table 1: Title Suggest instead of ” Clinical characteristics and laboratory test results at the first prenatal visit and OGTT result at 24-28 gestational weeks in pregnancy women with and without gestational diabetes mellitus (GDM) in the training cohort and validation cohort” - should be simplified to “Clinical characteristics and laboratory test results in pregnant women with and without gestational diabetes mellitus (GDM) in the training and validation cohort” Also add n=XX under the column headings

*Ans*: We have revised the table 1 according to your suggestion.

In Result (page 12, line 190):

**Table 1.** **Clinical characteristics and laboratory test results in pregnant women with and without gestational diabetes mellitus (GDM) in the training and validation cohort.**

|  | The training cohort | | | The validation cohort | | |
| --- | --- | --- | --- | --- | --- | --- |
| Baseline characteristics | Non-GDM | GDM | P value | Non-GDM | GDM | P value |
|  | N=437 (85.4 %) | N=75 (14.6 %) |  | N=125 (80.2 %) | N=26 (17.2 %) |  |
| Age (years) | 33.5 (4.1) | 35.1 (4.1) | **0.002** | 33.3 (3.6) | 33.8 (2.9) | 0.55 |
| Age≥35 (N, %) | 190 (43.5 %) | 42 (56 %) | **0.04** | 46 (36.8 %) | 11 (42.3 %) | 0.32 |
| Nulliparous (N, %) | 142 (32.5 %) | 26 (34.7 %) | 0.68 | 46 (36.8 %) | 13 (50 %) | 0.48 |
| Gestational age at the FPV (weeks) | 10.1 (1.9) | 10.3 (2.2) | 0.81 | 10 (1.5) | 9.9 (1.5) | 0.58 |
| Family history of DM (N, %) | 96 (22 %) | 26 (34.7 %) | **0.02** | 21 (16.8 %) | 5 (19.2 %) | 0.81 |
| History of PCOS | 16 (3.7 %) | 3 (4 %) | 0.89 | 7 (5.6 %) | 3 (11.5 %) | 0.27 |
| History of macrosomia | 2 (0.5 %) | 2 (2.7 %) | 0.05 | 0 | 0 | - |
| Pre-pregnancy BW (kg) | 55.3 (8.4) | 57.8 (14) | **0.03** | 55.2 (8.1) | 60.2 (8.5) | **0.005** |
| Pre-pregnancy BMI (kg/m2) | 21.9 (3.3) | 22.9 (5.3) | **0.02** | 21.9 (3.2) | 23.7 (3.5) | **0.02** |
| GWG at 24-28 gestational weeks (kg) | 6.3 (3.7) | 6.3 (2.7) | 0.4 | 6.1 (3.2) | 6.2 (2.3) | 0.92 |
| Laboratory test results at the first prenatal visit | | | |  |  |  |
| FPG (mg/dL) | 81.1 (5) | 88 (7.4) | **<0.001** | 82.8 (4.7)* | 90.9 (7.2) | **<0.001** |
| HbA1c (%) | 5.2 (0.2) | 5.4 (0.3) | **<0.001** | 5.1 (0.3)* | 5.3 (0.3) | **<0.001** |
| Glucose level during OGTT at 24-28 gestational weeks | | | |  |  |  |
| FPG during OGTT (mg/dL) | 77.9 (5) | 82.3 (6.9) | **<0.001** | 78.8 (5) | 85.2 (5.4) | **<0.001** |
| 1hPG (mg/dL) | 128.2 (24.3) | 164.7 (29.5) | **<0.001** | 127.6 (27.3) | 149 (34.3)**†** | **<0.001** |
| 2hPG (mg/dL) | 109.9 (19.9) | 150.2 (29.4) | **<0.001** | 108.6 (21.3) | 135.6 (39.7) | **<0.001** |

Mean (standard deviations) or N (%) were shown.

BMI, body mass index; BW, body weight; DM, diabetes mellitus; FPG, fasting plasma glucose; FPV, first prenatal visit; GDM, gestational diabetes mellitus; GWG, gestational weight gain; HbA1c, hemoglobin A1c; OGTT, oral glucose tolerance tests; FPG during OGTT, fasting plasma glucose during oral glucose tolerance tests; PCOS, Polycystic ovary syndrome; 1hPG, 1-hour plasma glucose during oral glucose tolerance tests; 2hPG, 2-hour plasma glucose during oral glucose tolerance tests.

* p<0.05 vs. women without GDM in the training cohort.

† p<0.05 vs. women with GDM in the training cohor

5. From table 1 – you appropriately highlight the small differences in blood sugar results between the validation and derivation cohort. This should be mentioned as a possible limitation to the validation portion of the analysis.

*Ans*: We have revised the manuscript according to your suggestion.

In Discussion (page 25, line 357-362): **Besides, there were some small differences, such as FPG at the FPV, HbA1c, and 1-hour plasma glucose during OGTT, between women with or without GDM in the training cohort and in the validation cohort. Although the differences between these groups were small, those differences still may lead to the different performance. It should be noted when interpreting the result.**

6. The prevalence of GDM is quite high – overall 14.6%. Does this seem reasonable for a universally tested Taiwanese population? If not – please address.

*Ans*: The International Association of the Diabetes and Pregnancy Study Group (IADPSG) criteria for GDM are associated with higher prevalence than the two-step method. In our previous study (J Diabetes Investig, 2016. 7(1): p. 121-126.), we have shown that adopting the IADPSG criteria increased the prevalence of GDM to 13.44%, compared with 2.59% by the Carpenter and Coustan (C&C) criteria. In another study by another group in Taiwan (PLoS One, 2015. 10(3). e0122261), the results showed that the incidence of GDM increased from 4.6% using the two-step method to 12.4% using the IADPSG criteria. Therefore, we think the prevalence of GDM in our study is reasonable by the IADPSG criteria in Taiwanese population.

7. The authors state in the discussion that “the algorithms used to screen GDM proposed in the study are simple, practical, and can be used clinically”. This is true but it needs to be mentioned that it requires that all women have a fasting glucose test early in pregnancy. This is often difficult logistically and unpleasant for women that are likely experiencing nausea and vomiting. This can be mentioned as a drawback to this strategy.

*Ans*: We have revised the manuscript according to your suggestion.

In Discussion (page 24-25, line 351-357): Secondly, pregnant women with FPG at FPV ≥ 92 mg/dl and <126 mg/dl were diagnosed as GDM according to the IADPSG guidelines, in addition to the criteria by the results of OGTT at 24-28th gestational weeks. **Since the diagnostic criteria for GDM varied in different countries, we have to emphasize that the conclusions of the present study are only applicable when GDM is diagnosed by the IADPSG criteria, which requires that all women have a fasting glucose test early in pregnancy. This may be difficult logistically and unpleasant for women.**

8. The discussion would usually start with a summary statement of the major findings. This is not the case here.

*Ans*: We have added a paragraph according to your suggestion.

In Discussion (page 21, line 284-291): **In the present study, we found that the incidence of GDM increased with age. The "FPG at the FPV" algorithm could reduce OGTT%** **to 68.8% with the FPG cutoff at 79 mg/dl. The "age plus FPG at the FPV" algorithm, with the cutoff of 114, further reduced OGTT% to 58.3%, with the sensitivity of 90.7% (9.3% GDM missed) and the specificity of 100%. These findings were replicated in the validation cohort. Besides, we also shown that when the percentage of women ≥35 years was between 30% and 60%, the screening algorithm with an “age plus FPG at the FPV” cutoff could reduce OGTT%, compared with the screening algorithm using FPG at FPV alone.**

9. Finally, I need to come back to the statement that there are no false positives and thus the specificity is 100%. See 2X2 table for the screening strategy of FPG at FPV using a threshold of 79 in all women with FPG <92 at FPV and using the values from S2 Fig:

|  | GDM+ | GDM- |  |
| --- | --- | --- | --- |
| FPG <79 | 6 | 125 | 131 |
| FPG ≥79 | 40 | 312 | 352 |
|  | 46 | 437 | 483 |

Sensitivity: 40/46= 86.9%

Specificity: 125/437 = 28.6%

NPV=125/131 = 95.4%

PPV= 40/352 = 11.4%

FPR: 312/437 = 71.4%

If you include those with FPG at FPV ≥ 92 results are as follows:

|  | GDM+ | GDM- |  |
| --- | --- | --- | --- |
| FPG < 79 | 6 | 125 | 131 |
| FPG ≥ 79 | 69 | 312 | 381 |
|  | 75 | 437 | 512 |
|  |  |  |  |
| Sens | 0.92 |  |  |
| Spec | 0.29 |  |  |
| NPV | 0.95 |  |  |
| PPV | 0.18 |  |  |
| FPR | 0.71 |  |  |

Thus, the statement (throughout the paper) that specificity is 100% is misleading. When everyone does FPG at FPV and ALL go on to have an OGTT (unless diagnosed by the FPG at FPV) then of course sensitivity and specificity are 100% but this type of strategy constitutes universal testing. In the model you are suggesting, only those with FPG <92 and greater than 79 would go on to have an OGTT. Most of those that screen “positive” (FPG >79) and thus have an OGTT do not have GDM and thus the PPV is low. Conversely, the majority of those without GDM screen positive at the FPV (312/437) and thus the specificity is 28.6% (FPR 71.4%). The low specificity is to be expected and is not necessarily problematic as this strategy still allows significantly fewer women to undergo a GTT at 24-28 weeks. Unless I am completely misunderstanding your explanation – this issue of specificity needs to be corrected throughout the paper.

*Ans*: Thanks for your detailed explanation. The key point is that we tested the performance of the whole algorithm, including FPG at the FPV ≥92 mg/dl, OGTT in women with FPG at the FPV ≥ 79 mg/dl, and exclusion of GDM in women with FPG at the FPV < 79 mg/dl, instead of testing the performance of the FPG at the FPV cutoff only. By the algorithm, GDM would not be diagnosed if the FPG at the FPV was ≥ 79 mg/dl. Instead, these women would receive OGTT to have a final diagnosis. Therefore, we also included OGTT% as another indicator for the performance of the algorithm.

To illustrate the concept clearer, we have made a 2x2 table for the “algorithm of FPG at the FPV” (S3 Table, shown below). In this algorithm, GDM could only be diagnosed by either FPG at FPV ≥ 92 mg/dl or the results of OGTT when FPG ≥ 79 mg/dl. Since these two criteria were gold standard method (ie. the IADPSG criteria), there would not be false positives (S2 Fig (A)), and the specificity would always be 100%.

We agree with you that this should be explained further. Therefore, we have added S3 Table. With S2 Fig and S3 Table, we believe that the calculation of the performance will be clearer.

In Results (page 16, line 242-244): The calculation of the sensitivity and the specificity for "the FPG at the FPV algorithm" with the cutoff at 79 mg/dl were illustrated in the S3 table. The numbers were derived from S2 Figure (A).

S3 Table. The 2x2 table illustrating the calculation of the sensitivity and the specificity for "the FPG at the FPV algorithm" with the cutoff at 79 mg/dl.

|  | GDM + by the IADPSG criteria | GDM- by the IADPSG criteria | Total |
| --- | --- | --- | --- |
| GDM diagnosed by the algorithm | 69 | 0 | 69 |
| GDM excluded by the algorithm | 6 | 437 | 443 |
| Total | 75 | 437 | 512 |
| Sensitivity | 69/75 = 0.92 |  |  |
| Specificity | 437/437 = 1.00 |  |  |

S2 Fig. Algorithms to screen gestational diabetes mellitus (GDM) by (A) fasting plasma glucose (FPG) at the first prenatal visit (FPV) and (B) age plus FPG at the FPV. The number of pregnant women in different paths are shown. The unit for age is years and the unit for FPG is mg/dL. OGTT, oral glucose tolerance tests; GDM excluded, pregnant women who were diagnosed as not having GDM; GDM diagnosed, pregnant women who were diagnosed as GDM.
